# Nuclear Quantum Effects on the Equation of State of Water: Insights from the Potential Energy Landscape Formalism

**DOI:** 10.1021/acs.jctc.5c02151

**Published:** 2026-03-20

**Authors:** Ali Eltareb, Gustavo E. Lopez, Nicolas Giovambattista

**Affiliations:** † Department of Physics, 2037 Brooklyn College of the City University of New York, Brooklyn, New York 11210, United States; ‡ Ph.D. Program in Physics, 14772 The Graduate Center of the City University of New York, New York, New York 10016, United States; § Department of Chemistry, Lehman College of the City University of New York, Bronx, New York 10468, United States; ∥ Ph.D. Program in Chemistry, 14772 The Graduate Center of the City University of New York, New York, New York 10016, United States

## Abstract

We apply the potential energy landscape (PEL) formalism for quantum liquids, together with path-integral (PI) computer simulations, to derive the equation of state (EOS) for both equilibrium and supercooled water over a wide range of temperatures and pressures. The PEL-EOS for water, which includes nuclear quantum effects (NQE), is in very good agreement with the PI computer simulations, particularly in the proximity of water’s liquid–liquid critical point (LLCP). Relative to the classical case, including NQE shifts the overall phase diagram of water toward lower temperatures and slightly lower pressures. In particular, the LLCP temperature and pressure are shifted by Δ*T*_*c*_ ≈ 18 K and Δ*P*_*c*_ ≈ 49 MPa, with a minor change in the LLCP density, Δρ_*c*_ ≈ 0.002 g/cm^3^. These values of (Δ*P*_*c*_, Δ*T*_*c*_, Δρ_*c*_) represent, approximately, a maximum shift for the location of the LLCP for H_2_O due to isotope substitution (H_2_O → D_2_O → T_2_O). Additionally, NQE also affect the shape of the density and LL spinodal lines in the P-T plane. The PEL of (q-TIP4P/F) water is Gaussian, allowing for the evaluation of the configurational entropy *S*_IS_(*T*, *V*) and Kauzmann temperature, *T*_*K*_(*V*). NQE reduce the *T*_*K*_(*V*) of water by 5–20 K depending on the density, consistent with the observed increase in water diffusion coefficient *D* at low temperatures upon the inclusion of quantum fluctuations. Notably, the Adam–Gibbs relationship, which relates *D* and *S*_IS_, holds remarkably well at all densities studied. From the perspective of the PEL formalism, NQE primarily modify the curvature of water’s PEL basins while the corresponding IS remain unchanged, isomorphic to the IS of classical water. The PEL-based approach employed in this work is versatile and physically intuitive, suitable for calculating the free energy and EOS of quantum liquids beyond water.

## Graphical abstract

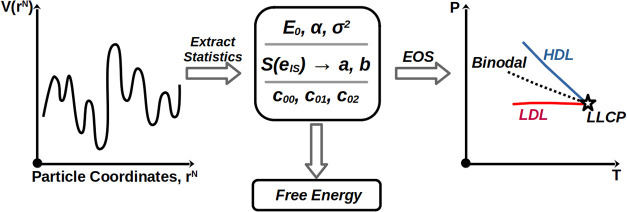

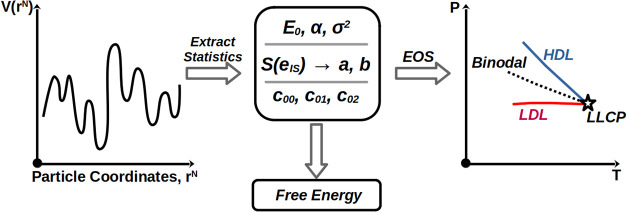

## 1 Introduction

Water is an ubiquitous liquid in Earth, playing a fundamental role in chemistry, biology, and environmental science.^1–4^ Its complex behavior across various states—including ice, equilibrium and supercooled liquid, and glassy phases—exhibit numerous anomalies.^5–10^ For example, in its crystalline form, water manifests in 19 distinct ice varieties.^11–13^ In both the equilibrium and supercooled liquid states, water displays maxima in density, isobaric specific heat, and compressibility, alongside a liquid–liquid phase transition (LLPT) characterized by an associated liquid–liquid critical point (LLCP).^14–17^ Moreover, in its glass state, water can form two types of amorphous ice at approximately *P* < 1000 MPa, which are separated by an apparent first-order phase transition.^18^ Recent experiments^14,15,19–22^ and simulations^23–35^ provide strong support for water’s LLPT/LLCP, with estimates placing the LLCP at *P*_*c*_ ≈ 100–200 MPa and *T*_*c*_ ≈ 200 K.^22,31,36^

From a computational standpoint, our understanding of water’s phase behavior in the liquid and glassy states (across wide-ranging temperatures and pressures) largely derives from classical molecular dynamics (MD) simulations, which treat oxygen and hydrogen atoms as point-like interacting sites. Accordingly, in these computational studies, nuclear quantum effects (NQE) are not properly accounted for since the delocalization of the O and H atoms is neglected. This can be problematic; the atoms delocalization can weaken the hydrogen bond and alter the structural, dynamic, and thermodynamic properties of water.^37–40^ For example, the melting temperature of H_2_O and D_2_O differ by 4 K^37^ and the glass transition temperature of D_2_O is ≈10 K higher than that of H_2_O.^41^ Computer simulations that include NQE such as path-integral (PI) simulations, suggest that NQE shift the LLPT/LLCP toward lower temperatures and pressures.^24^ However, characterizing the influence of NQE on water’s phase behavior using PI simulations, at low temperatures and in the proximity of the LLPT/LLCP, has been computationally challenging due to water’s slow dynamics and large equilibration times. Motivated by such limitations, in this work we introduce an innovative approach that combines PI computer simulations with the potential energy landscape (PEL) formalism to derive an analytical expression for the Helmholtz free energy of water. This expression enables straightforward calculations of the equation of state (EOS), which can be utilized to study the LLPT/LLCP in water while accounting for NQE, at conditions where PI simulations become computationally very expensive. Previous applications of this methodology, together with classical molecular dynamics simulations, have successfully identified the LLCP location for the classical SPCE, TIP4*P*/2005, and q-TIP4P/F water models^25,29,42,43^ and the DP_MBpol machine learned potential.^44^ By extending the PEL formalism for quantum liquids with PI simulations, we demonstrate the effectiveness of this approach in incorporating NQE for water.

The PEL formalism is a theoretical approach based on statistical mechanics that has been extensively applied to study complex systems, such as liquids and glasses. Briefly, for a *classical* system composed of *N* atoms, the PEL is the hyper-surface in (3*N* + 1)-dimensional space defined by the potential energy of the system $V({\mathrm{r⃗}}_{1},{\mathrm{r⃗}}_{2},\cdot \cdot \cdot {\mathrm{r⃗}}_{N})$ as a function of the system’s 3*N* degrees of freedom, $\left\{{\mathrm{r⃗}}_{1},{\mathrm{r⃗}}_{2},\cdot \cdot \cdot {\mathrm{r⃗}}_{N}\right\}$ . In this framework, the PEL is partitioned into basins, each basin being associated with a PEL local minimum or inherent structure (IS).^45–48^ The dynamics of the system can be envisioned as the movement of a representative point navigating from one basin of the PEL to another, overcoming potential energy barriers with time. Notably, under simplified assumptions, the PEL framework provides a formal expression for the Helmholtz free energy of the system that depends on a limited set of topographic properties of the PEL, including the number of IS accessible at the given working conditions, the shapes of these basins, and their corresponding (IS) energies. Such topographic properties of the PEL can be sampled using computer simulations from which the Helmholtz free energy of the system, and all its thermodynamic properties, can be evaluated.^25,29,42,43,49–55^ Relevant to this work, we note that recent investigations have advanced the PEL formalism to study quantum systems, utilizing path-integral statistical mechanics, where atoms are represented as ring-polymers.^56,57^ While classical and quantum PEL formulations are conceptually similar, contrary to the classical case, the PEL of quantum liquids is temperature-dependent. Despite its theoretical promise, practical applications of the PEL formalism to quantum liquids remain limited. In this study, we employ the PEL formalism for quantum liquids to compute the EOS of water with NQE included, utilizing PI simulations based on the q-TIP4P/F water model.^58^ Our PEL-EOS achieves excellent concordance with PI simulation data across a wide temperature and volume range, allowing us to pinpoint the location of the LLCP for this model. Moreover, we investigate key properties of q-TIP4P/F water, including the configurational entropy and its relationship to water dynamics via the Adam–Gibbs relationship.

The structure of this manuscript is as follows. Section 2 presents a brief introduction to the PEL formalism for quantum liquids, including the Gaussian and harmonic approximations, and presents the corresponding PEL-EOS. Computational methodologies are discussed in Section 3, while Section 4 details the results from our PI simulations and PEL analysis of q-TIP4P/F water. Finally, a summary and discussion of our findings are provided in Section 5.

## 2 Equation of State for Quantum Liquids Based on the Potential Energy Landscape Formalism

The PEL formalism for quantum liquids is based on the PI formulation of statistical mechanics, where the system of interest (e.g., a liquid) is mapped onto a classical system composed of ring-polymers.^59,60^ It is the PEL of such a ring-polymer system (RP-PEL) which is associated with the quantum liquid.^54,57,61^ Using the RP-PEL, one can naturally extend the PEL formalism, originally developed for classical liquids, to liquids that obey quantum mechanics (see refs 54,57 for details).

The PEL formalism provides a formal expression for the Helmholtz free energy *F*(*N*, *V*, *T*) of a quantum liquid.^54,57,61^ For the case of a water model such as the q-TIP4P/F model, where the interatomic interactions depend only on the H and O coordinates, the PEL formalism leads to the following expression,

$$F(N,V,T)=E_{\mathrm{IS}}(N,V,T)-TS_{\mathrm{IS}}(N,V,T,E_{\mathrm{IS}})+F_{\mathrm{vib}}(N,V,T,E_{\mathrm{IS}})$$ 1

where *N* is the number of molecules, and the quantities (i) *E*_IS_, (ii) *S*_IS_, (iii) *F*_vib_ are defined below. Note that eq 1 also applies to classical liquids^46,48,62^ although, in the classical case, *S*_IS_ = *S*_IS_(*N*, *V*, *E*_IS_) since the PEL of classical liquids are *T*-independent, while the PEL of quantum liquids (RP-PEL) are not.

(i) *E*_IS_(*N*, *V*, *T*) is the average IS energy of the system, and is the solution to the following equation,

1−T(∂SIS(N,V,T,eIS)∂eIS)N,V,T+(∂Fvib(N,V,T,eIS)∂eIS)N,V,T=0 2

for *e*_IS_ = *E*_IS_. In computational studies, *E*_IS_(*N*, *V*, *T*) is identified with the average IS energy sampled by the system at the given working conditions, (*N*, *V*, *T*); briefly, *E*_IS_(*N*, *V*, *T*) = ⟨*e*_IS_⟩_*N*,*V*,*T*_.

(ii) *S*_IS_(*N*, *V*, *T*, *e*_IS_) is the configurational entropy of the quantum liquid/ring-polymer system defined as

$$S_{\mathrm{IS}}(N,V,T,e_{\mathrm{IS}})=k_{B}\ln[\Omega_{\mathrm{IS}}(N,V,T,e_{\mathrm{IS}})]$$ 3

where Ω_IS_(*N*, *V*, *T*, *e*_IS_) is the number of IS available in the RP-PEL with energy *e*_IS_. Most classical MD and PI computer simulations of water using different models, including the q-TIP4P/F model, indicate that the PEL/RP-PEL of water is Gaussian, i.e., the corresponding distribution of IS energies can be approximated by a Gaussian distribution,^25,29,42,43,52,54,57,61,63^

$$\Omega_{IS}(N,V,e_{\mathrm{IS}})=\frac{1}{\sqrt{2\pi \sigma^{2}}}e^{\alpha N}e^{-(e_{\mathrm{IS}}-E_{0}{)}^{2}/2\sigma^{2}}$$ 4

where (α, σ^2^, *E*_0_) are PEL-variables that depend only on *V*.^25,48,61^ It follows from eq 3 that in this case, *S*_IS_ is *T*-independent and is given by

$$S_{\mathrm{IS}}(N,V,e_{\mathrm{IS}})\approx k_{B}\left[\alpha N-\frac{(e_{\mathrm{IS}}-E_{0}{)}^{2}}{2\sigma^{2}}\right]$$ 5

(iii) *F*_vib_(*N*, *V*, *T*, *e*_IS_) is the vibrational Helmholtz free energy of the system and it is the contribution to the free energy *F*(*N*, *V*, *T*) due to the exploration (by the quantum liquid/ring-polymer system) of the RP-PEL basins with energy *e*_IS_. A formal definition of *F*_vib_(*N*, *V*, *T*, *e*_IS_) is provided within the PEL formalism; see refs 54,57,61 . It can be shown that, if the basins of the RP-PEL are parabolic (harmonic approximation) then^48,54^

$$F_{\mathrm{vib}}(N,V,T,e_{\mathrm{IS}})=F_{\mathrm{vib}}^{\mathrm{harm}}(N,V,T,e_{\mathrm{IS}})=9Nn_{b}k_{B}T\;\ln(\beta A_{0})+k_{B}TS(N,V,T,e_{\mathrm{IS}})$$ 6

where 9*N* is the total number of degrees of freedom in the system (water), and *n*_*b*_ is the number of beads per ring-polymer;^54,56,57^ *A*_0_ is an arbitrary constant that makes the argument in the ln(···) unit-less (we adopt the standard value used in PEL studies of water, *A*_0_≡ 1 kJ/mol). In eq 6, $S(N,V,T,e_{\mathrm{IS}})$ is the basin shape function;^48^ $S(N,V,T,e_{\mathrm{IS}})$ quantifies the average local curvature of the RP-PEL basins with energy *e*_IS_, about the corresponding IS. Specifically,

$$S(N,V,T,e_{\mathrm{IS}})\approx {\left\langle \sum_{i=1}^{9Nn_{b}-3}\ln\left(\frac{\hbar \omega_{i}(N,V,T,e_{\mathrm{IS}})}{A_{0}}\right)\right\rangle }_{e_{\mathrm{IS}}}$$ 7

In this expression, {ω_*i*_^2^}_*i*=1,2,···_ are the eigenvalues of the mass-weighted Hessian matrix of the ring-polymer system evaluated at the IS of the RP-PEL with energy e_IS_, at the given (*N*, *V*, *T*).^25,43,48,54,57^ The ⟨···⟩_*e*_IS__ indicates an average over all IS of the RP-PEL with energy *e*_IS_. Note that since the RP-PEL associated with the quantum liquid is *T*-dependent, the quantities {ω_*i*_^2^}_*i*=1,2···_ also vary with *T*. Since an IS is a potential energy minimum of the ring-polymer system, all the 9*Nn*_*b*_ eigenvalues of the mass-weighted Hessian matrix are greater than zero, except for the three eigenvalues associated with the system’s center of mass motion which are zero. As will be shown below, and consistent with previous PI computer simulations (including q-TIP4P/F water),^54,57,61^ we find that for all isochores examined,

$$S(N,V,T,e_{\mathrm{IS}})=a(N,V,T)+b(N,V,T)e_{\mathrm{IS}}$$ 8

where *a* and *b* are coefficients that depend on (*N*, *V*, *T*).

Classical MD and PI computer simulations of water based on different models, including the q-TIP4P/F model, show that the PEL/RP-PEL of water is not harmonic and hence, anharmonic corrections need to be included. In this work, we follow ref 53,61 and model the anharmonic contributions as

$$F_{\mathrm{vib}}(N,V,T,e_{\mathrm{IS}})=F_{\mathrm{vib}}^{\mathrm{harm}}(N,V,T,e_{\mathrm{IS}})+F_{\mathrm{vib}}^{\mathrm{anh}}(N,V,T,e_{\mathrm{IS}})$$ 9

where *F*_vib_^harm^(*N*, *V*, *T*, *e*_IS_) is given by eq 6 and

$$\beta F_{\mathrm{vib}}^{\mathrm{anh}}(N,V,T,e_{\mathrm{IS}})={\overset{∼}{B}}_{0}(N,V,T)+{\overset{∼}{B}}_{1}(N,V,T)e_{\mathrm{IS}}$$ 10

In ref 53, it is found that *B̃*_1_ ≈ 0 for q-TIP4P/F water at ρ = 1.0 g/cm^3^ and approximately, *T* < 280 K. As we will show here, *B̃*_1_ ≈ 0 for all the isochores studied (for approximately *T* < 280 K). Moreover, consistent with ref 53, the results from our PI computer simulations can be modeled by assuming that

$${\mathrm{B̃}}_{0}(N,V,T)=c_{0,0}(N,V)+c_{0,1}(N,V)T+c_{0,2}(N,V)T^{2}$$ 11

where *c*_0,*j*=0,1,2_ are *T*-independent coefficients.

Using eqs 9, 6, and 10, the final expression for the Helmholtz free energy of water, based on the PEL formalism and including NQE, is given by

F(N,V,T)=EIS(N,V,T)−TSIS(N,V,eIS)+9NnbkBTln(βA0)+kBTS(N,V,T,EIS)+1β[B∼0(N,V,T)+B∼1(N,V,T)EIS(N,V,T)] 12

This expression follows from eq 1, assuming that the RP-PEL associated with water is anharmonic (eq 9), with anharmonic corrections given by eqs 10 and 11. In eq 12, *S*_IS_ and S are given by eqs 5 and 8, respectively; as we will show below, both expressions are consistent with our PI computer simulations. In addition, one can show from eq 2 that the IS energy is given by (see refs 53,54 )­

$$E_{IS}\left(N,V,T\right)=E_{0}\left(V\right)-\sigma^{2}(V)\left[\beta +b\left(N,V,T\right)+{\overset{∼}{B}}_{1}\left(N,V,T\right)\right]$$ 13

Note that if *B̃*_1_ = 0, then eq 13 reduces to the well-known expression of *E*_IS_ for a Gaussian and harmonic PEL,

$$E_{\mathrm{IS}}^{\mathrm{harm}}(N,V,T)=E_{0}(V)-\sigma^{2}(V)[\beta +b(N,V,T)]$$ 14

The pressure equation-of-state (EOS) of water, based on the PEL formalism (briefly, PEL-EOS), can be obtained from eq 12 using the thermodynamic relation, *P* = −(∂*F*/∂*V*)_*N*,*T*_. From eqs 12, 5, 8, 11, and 13, and for the case where *B̃*_1_ ≈ 0, one obtains the following PEL-EOS,

$$P\left(N,V,T\right)=P_{-1}\left(V\right)T^{-1}+P_{0}\left(N,V,T\right)+P_{1}\left(N,V,T\right)T+P_{2}\left(N,V\right)T^{2}+P_{3}\left(N,V\right)T^{3}$$ 15

where

$$P_{-1}\left(V\right)=\frac{1}{2k_{B}}\frac{d\sigma^{2}\left(V\right)}{dV}$$ 16

$$P_{0}\left(N,V,T\right)=-\frac{\partial }{\partial V}{\left[E_{0}\left(V\right)-b\left(N,V,T\right)\sigma^{2}\left(V\right)\right]}_{N,T}$$ 17

$$P_{1}\left(N,V,T\right)=k_{B}\frac{\partial }{\partial V}{\left[N\alpha \left(V\right)-a\left(N,V,T\right)-b\left(N,V,T\right)E_{0}\left(V\right)+\frac{b^{2}\left(N,V,T\right)\sigma^{2}\left(V\right)}{2}-c_{0,0}\left(N,V\right)\right]}_{N,T}$$ 18

$$P_{2}\left(N,V\right)=-k_{B}{\left(\frac{\partial c_{0,1}\left(N,V\right)}{\partial V}\right)}_{N}$$ 19

$$P_{3}\left(N,V\right)=-k_{B}{\left(\frac{\partial c_{0,2}\left(N,V\right)}{\partial V}\right)}_{N}$$ 20

It follows that the pressure PEL-EOS depends on the eight PEL variables {α­(*V*), *E*_0_(*V*), σ^2^(*V*), *a*(*N*, *V*, *T*), *b*(*N*, *V*, *T*), *c*_0,0_(*N*, *V*), *c*_0,1_(*N*, *V*), *c*_0,2_(*N*, *V*)}. These PEL variables depend on the system (water model) studied and hence, they must be obtained from the PI computer simulations. The remarkable success of the PEL formalism is that for water (and other liquids), it is possible to find numerically the set of variables {α­(*V*), *E*_0_(*V*), σ­(*V*)^2^, *a*(*N*, *V*, *T*), *b*(*N*, *V*, *T*), *c*_0,0_(*N*, *V*), *c*_0,1_(*N*, *V*), *c*_0,2_(*N*, *V*)} that, combined with the PEL expressions provided in this section, can fit the data from the PI computer simulations. We stress that the pressure PEL-EOS for classical water (and liquids in general), based on the Gaussian approximation of the PEL, is similar to eq 15. However, for quantum water, the coefficients {*P*_*j*_}_*j*=0,1_ may depend (implicitly) on *T* while, in the classical case, all coefficients are temperature-independent.^25,29,42,43,48–50^

## 3 Computational Details

We perform ring-polymer molecular dynamics (RPMD) simulations of a system composed of *N* = 512 water molecules placed in a cubic box. The system is periodic along the three dimensions and the water molecules are represented using the flexible q-TIP4P/F model.^58^ The q-TIP4P/F water model reproduces several properties of liquid water at *P* = 0.1 MPa in the equilibrium and supercooled regime relatively well.^64^ However, as for most water models, the q-TIP4P/F water model underestimates many of the thermodynamic response functions in the supercooled regime at very low temperatures (*T* < 240 K), including the magnitude of the maxima in the isothermal compressibility and isobaric heat capacity observed in experiments at *T* ≈ 230 K.^14,17^ The same computational techniques used in our previous studies, refs 25,40,57,65 , are employed here; we refer the reader to those works for a detailed discussion. Briefly, RPMD simulations are performed using the OpenMM software package (version 7.4.0) over a wide range of temperatures (200 ≤ *T* ≤ 300 K) and volumes (0.92 ≤ ρ ≤ 1.40 g/cm^3^). The temperature of the system is maintained using the stochastic (local) path-integral Langevin equation (PILE) thermostat,^66^ where the thermostat collision frequency parameter γ = 0.1 ps^–1^. The simulations time step is *dt* = 0.25 fs, and the number of beads per ring-polymer is *n*_*b*_ = 32. The value *n*_*b*_ = 32 is large enough for most thermodynamic properties of q-TIP4P/F water to converge^24,38,64^ and it is a standard value employed in the study of liquid water. The short-range (Lennard–Jones pair potential) interactions are calculated using a cutoff distance *r*_*c*_ = 1.0 nm, and the long-range electrostatic interactions are calculated using the reaction-field technique^67^ with the same cutoff distance *r*_*c*_. For each temperature and volume, the system is equilibrated for 1–50 ns, followed by production runs of 1–100 ns, depending on the temperature of the system; see Table S1 in the Supporting Information (SM) for more details.

During the RPMD simulations at a given (*T*, *V*), we save 25 independent configurations equally spaced in time. These configurations are subjected to potential energy minimizations using the L-BFGS-B algorithm.^68^ The IS and average IS energy, *E*_IS_, are obtained directly from the minimization procedure. Importantly, in all cases, we find that the ring-polymers associated with water O and H atoms collapse at the IS, consistent with previous computational studies of water and monatomic systems^53,54,56,57,61^ [specifically, at the IS (after the potential energy of the system is minimized), all the beads belonging to a given ring-polymer are found located at the same position in real space]. As explained in refs 53,54,57 , the collapse of the ring-polymers at the IS of the RP-PEL allows one to calculate the normal mode vibrational frequencies at the IS from classical MD simulations. Specifically, following refs 53,57 , the vibrational density of states of quantum q-TIP4P/F water at the IS are computed by first evaluating the eigenvalues of mass-weighted Hessian of *classical* q-TIP4P/F water, using configurations obtained from classical MD simulations (see refs 25,54,57 ). In order to do this, classical MD simulations are performed at the same state points (*N* = 512, *V*, *T*) studied using the RPMD simulations. For each state point (*T*, *V*), we extract 25 equally spaced configurations from which the IS are obtained. For each IS, we evaluate the mass-weighted Hessian matrix elements, and the corresponding eigenvalues, from which the normal-mode frequencies {ω_*i*,0_}_*i*=1,2,···,(9*N*−3)_ are obtained; see ref. 53,57 The normal-mode frequencies of the quantum q-TIP4P/F water are obtained analytically from the set {ω_*i*,0_}_*i*=1,2,···,(9*N*−3)_ in a straightforward manner (see refs 53,54,57 and discussion below).

## 4 Results

The main goal of this work is to show that the pressure PEL-EOS, eq 15, is in good agreement with results from PI computer simulations of q-TIP4P/F water over a wide range of densities and temperatures. To do so, we first evaluate the PEL variables {α­(*V*), *E*_0_(*V*), σ^2^(*V*), *a*(*N*, *V*, *T*), *b*(*N*, *V*, *T*), *c*_0,0_(*N*, *V*), *c*_0,1_(*N*, *V*), *c*_0,2_(*N*, *V*)} from the PI computer simulations. While doing so, we validate eq 8 for S , and eq 13 for *E*_IS_ (Gaussian approximation) (Sec. [Sec sec4.1]); we also show that the anharmonic contributions to the RP-PEL associated with q-TIP4P/F water can be modeled using eq 10 (Sec. [Sec sec4.2]). In Sec. [Sec sec4.3], we compare the PEL-EOS with the results from PI computer simulations and show that, consistent with previous PI simulations, introducing NQE shift the phase diagram of supercooled water toward lower temperatures and pressures, including the LLCP. We conclude with a brief discussion on the configurational entropy of water and its relevance to water’s dynamics. Specifically, we show that the Adam–Gibbs relationship works remarkably well at all temperatures and densities studied (Sec. [Sec sec4.4]).

The procedure and technical details followed to evaluate the PEL variables {α­(*V*), *E*_0_(*V*), σ^2^(*V*), *a*(*N*, *V*, *T*), *b*(*N*, *V*, *T*), *c*_0,0_(*N*, *V*), *c*_0,1_(*N*, *V*), *c*_0,2_(*N*, *V*)} are explained in detail in our previous work, ref 53, where we evaluate these PEL variables for the isochore ρ = 1.00 g/cm^3^ using PI computer simulations of q-TIP4P/F water. Briefly, {α­(*V*), *E*_0_(*V*), σ^2^(*V*)} are taken from ref 25, where these quantities are obtained from *classical* MD simulations of q-TIP4P/F water. Indeed, as explained in ref 53, {α­(*V*), *E*_0_(*V*), σ^2^(*V*)} are not affected by NQE provided that the ring-polymers associated with the quantum liquid collapse at the IS of the RP-PEL (which we confirm in this work). The PEL variables {*a*(*N*, *V*, *T*), *b*(*N*, *V*, *T*)} are evaluated by calculating the shape function S using eqs 7 and 8. As explained in refs 54,57 , the vibrational normal-mode frequencies ${\left\{\omega_{i}\right\}}_{i=1,2...,\left(9Nn_{b}\right)}$ of the ring-polymer system (associated with the quantum liquid) are evaluated from classical MD simulations of q-TIP4P/F water. The PEL variables {*c*_0,0_(*N*, *V*), *c*_0,1_(*N*, *V*), *c*_0,2_(*N*, *V*)}, which quantify the anharmonicities of the PEL basins, are evaluated from the PI computer simulations (as explained below).

### 4.1 Shape Function and Gaussian Approximation

Figure 1a shows the shape function S (*e*_IS_) as a function of the energy *e*_IS_ of the IS available in the RP-PEL; results are included for selected temperatures, *T* = 210, 240, 280 K, and over a wide range of densities, ρ = 0.94–1.24 g/cm^3^. For all the *T* and *V* studied, we find that S is a linear function of *e*_IS_, validating eq 8 (*N* is constant) and consistent with previous PEL studies based on PI computer simulations.^25,42,43^ Interestingly, at a given *e*_IS_ and *temperature* (see, e.g, upper panel of Figure 1a), S­(*e*_IS_) increases slightly with increasing density. This implies that, at a given temperature, the basins of the RP-PEL at a given depth become slightly narrower upon compression. Similarly, at a given *e*_IS_ and *density* (see, e.g., black lines in the three panels of Figure 1a), S­(*e*_IS_) increases with increasing temperature. Accordingly, the basins of the PEL at a given depth also become narrower upon heating (at a given density).

**Figure 1:**
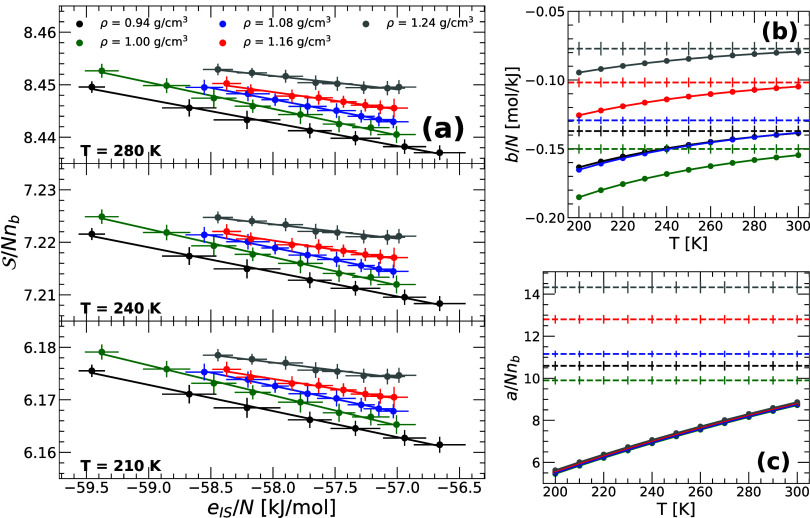
(a) Basin shape function, S , as a function of *e*_IS_ for selected temperatures and densities (*T* = 210, 240, 280 K and ρ = 0.94–1.24 g/cm^3^) obtained from PI computer simulations of q-TIP4P/F water. The solid lines are linear fits to the data based on eq 8. The corresponding fitting parameters *a* and *b* are shown in (b) and (c) [symbols and solid lines]. For comparison, also included are the (*T*-independent) values of *a* and *b* obtained from classical MD simulations of q-TIP4P/F water (dashed lines) reported in ref 25.

The PEL variables *a*(*V*, *T*) and *b*(*V*, *T*) (*N* is constant) resulting from the linear fits in Figure 1a are included in Figure 1b,c. Both *a*(*V*, *T*) and *b*(*V*, *T*) increase monotonically with increasing temperature along a given isochore. The dashed-lines in Figure 1b,c are the values of *a* and *b* for classical q-TIP4P/F water reported in ref 25. In this case, *a* and *b* are independent of temperature since for classical liquids, the PEL is *T*-independent.

The PEL-EOS, eq 15, is based on the assumption that (i) the RP-PEL associated with q-TIP4P/F water is Gaussian, with (ii) the parameters {α­(*V*), *E*_0_(*V*), σ^2^(*V*)} being identical to those for classical q-TIP4P/F water. To validate (i) and (ii), we include in Figure 2a the average IS energy *E*_IS_(*T*) obtained from the RPMD simulations (symbols) as a function of [β + *b*(*T*)] for selected isochores (the PEL variable *b*(*T*) is taken from Figure 1b). Also included in Figure 2a are the PEL predictions for *E*_IS_(*T*) given by eq 14 with the PEL variables *E*_0_(*V*) and σ^2^(*V*) obtained independently in ref 25 based on classical MD simulations (lines). Eq 14 is in very good agreement with the RPMD simulations at approximately *T* ≤ 280 K and for all densities studied indicating that, indeed, the RP-PEL associated with q-TIP4P/F water is Gaussian. Moreover, by comparing eqs 13 and 14, it follows that the PEL variable *B̃*_1_(*T*), and hence the anharmonic contributions to *E*_IS_ [given by (−σ^2^ *B̃*_1_)], are negligible. As shown in Figure 2b, *B̃*_1_(*T*) is very small, ranging from −0.01 to 0.02 mol/kJ, implying that |σ^2^ *B̃*_1_(*T*)| < 0.15 kJ/mol and hence, it represents a negligible contribution to *E*_IS_ (<−56 kJ/mol). The main points of Figure 2a are that (i′) the Gaussian approximation, holds for q-TIP4P/F water at all densities considered (at approximately *T* ≤ 280 K) with (ii′) the parameters obtained from classical MD simulations and (iii′) *B̃*_1_(*T*) ≈ 0.

**Figure 2:**
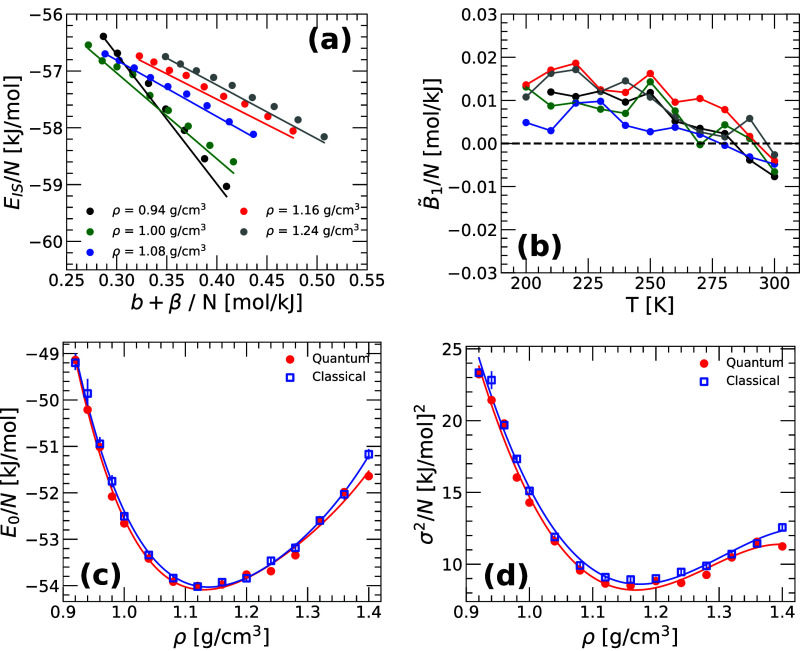
(a) Average IS energy *E*_IS_(*T*) as a function of [β + *b*(*T*)] for q-TIP4P/F water obtained from PI computer simulations. Results are for ρ = 0.94–1.24 g/cm^3^; *b*(*T*) is taken from Figure 1b. Solid lines correspond to the predictions for *E*_IS_(*T*) given in eq 14 based on the Gaussian approximation of the PEL and neglecting the anharmonic contribution. The PEL variables {*E*_0_(*V*), σ^2^(*V*)} used in eq 14 correspond to the classical values of q-TIP4P/F water reported in ref 25. Eq 14 is in very good agreement with the PI computer simulations. (b) The PEL variable ${\tilde{B}}_{1}(T)$ defined in eq 13, obtained from (a). ${\tilde{B}}_{1}(T)$ is negligible at all densities and temperatures considered implying that anharmonic contributions to *E*_IS_(*T*) are not relevant. (c) and (d) PEL variables *E*_0_ and σ^2^ as a function of density for q-TIP4P/F water. Blue squares are the parameters that define the straight lines in (a), taken from the classical MD simulations of ref^25^. For comparison, also included are the corresponding values obtained by *fitting* the *E*_IS_(*T*) for *T* ≤ 280 K [circles in (a)] using eq 14; see text.

Figure 2c,d show the PEL variables {*E*_0_(*V*), σ^2^(*V*)} that define the straight lines in Figure 2a (blue squares; taken from ref^25^. We also include in Figure 2c,d (red circles) the PEL variables {*E*_0_(*V*), σ^2^(*V*)} obtained by *fitting* the values of *E*_IS_(*T*) from the RPMD simulations at *T* ≤ 280 K (circles in Figure 2a) using eq 14 (assuming *B̃*_1_(*T*) ≈ 0). The values of {*E*_0_(*V*), σ^2^(*V*)} obtained from the PI and MD simulations practically overlap at all densities considered providing further support that, indeed, the distribution of IS energies in the PEL of (q-TIP4P/F) water (eq 4) is not affected by the inclusion of NQE. In other words, quantum fluctuations modify the curvatures of the PEL basins of water (Figure 1b,c) but do not alter the IS energy distribution, Ω_IS_(*N*, *V*, *T*, *e*_IS_), and therefore, *S*_IS_(*N*, *V*, *T*, *e*_IS_) (Figure 2a,c,d). Our results are consistent with previous PEL studies of monatomic model liquids as well as q-TIP4P/F water (limited to ρ = 1.00 g/cm^3^).^53,57,61^

### 4.2 Harmonic Approximation and Anharmonic Corrections

Next, we first show that the RP-PEL of q-TIP4P/F water is not harmonic, which is not surprising given that the PEL of classical water models are not harmonic either (Sec. [Sec sec4.2.1]).^25,42,43^ We then calculate the anharmonic contributions of q-TIP4P/F water (eq 10) from PI computer simulations and obtain the PEL variables {*c*_0,1_(*V*), *c*_0,2_(*V*)} (Sec. [Sec sec4.2.1]). The configurational entropy and the PEL variable *c*_0,0_(*V*) are evaluated in Sec. [Sec sec4.2.2].

#### 4.2.1 Vibrational Energy–Finding {*c*_0,1_(*V*), *c*_0,2_(*V*)}

The PEL formalism provides an expression for the total energy of a quantum liquid, *E*(*N*, *V*, *T*) = (∂(β*F*)/∂β)_*N*,*V*_. It can be shown that^53,54,57,61^

$$E(N,V,T)=E_{\mathrm{IS}}(N,V,T)+E_{\mathrm{vib}}(N,V,T)$$ 21

where *E*_vib_(*N*, *V*, *T*) is the vibrational energy of the corresponding ring-polymer system. Within the harmonic approximation of the PEL,^54,57^ one finds that

$$E_{\mathrm{vib}}(N,V,T)=E_{\mathrm{vib}}^{\mathrm{harm}}(N,V,T)=dn_{b}k_{B}T+{\left(\frac{\partial S}{\partial \beta }\right)}_{N,V,E_{\mathrm{IS}}}$$ 22

where *d* is the number of degrees of freedom in the system (*d* = 9*N* for q-TIP4P/F water). Instead, if anharmonic corrections are included, and modeled using eq 10, one can further show that

$$E_{\mathrm{vib}}(N,V,T)=E_{\mathrm{vib}}^{\mathrm{harm}}(N,V,T)+E_{\mathrm{vib}}^{\mathrm{anh}}(N,V,T,E_{\mathrm{IS}})$$ 23

where *E*_vib_^harm^(*N*, *V*, *T*) is given by eq 22, and

$$E_{\mathrm{vib}}^{\mathrm{anh}}(N,V,T,E_{\mathrm{IS}})={\left(\frac{\partial {\tilde{B}}_{0}}{\partial \beta }\right)}_{N,V}+{\left(\frac{\partial {\tilde{B}}_{1}}{\partial \beta }\right)}_{N,V}E_{\mathrm{IS}}$$ 24

In the case of q-TIP4P/F water, *B̃*_1_ ≈ 0. Hence, from eqs 11 and 24, it follows that

$$E_{\mathrm{vib}}^{\mathrm{anh}}(N,V,T,E_{\mathrm{IS}})=-k_{B}[c_{0,1}(N,V)T^{2}+2c_{0,2}(N,V)T^{3}]$$ 25

Figure 3a shows the *E*_vib_(*T*) of q-TIP4P/F water as a function of temperature for several densities. The circles correspond to the values of *E*_vib_(*T*) obtained from the PI computer simulations; the lines are the predictions from the PEL formalism with the harmonic approximation, eq 22. At all densities, *E*_vib_^harm^(*T*) overestimates *E*_vib_(*T*), confirming that the basins surrounding the IS of quantum water are anharmonic.

**Figure 3:**
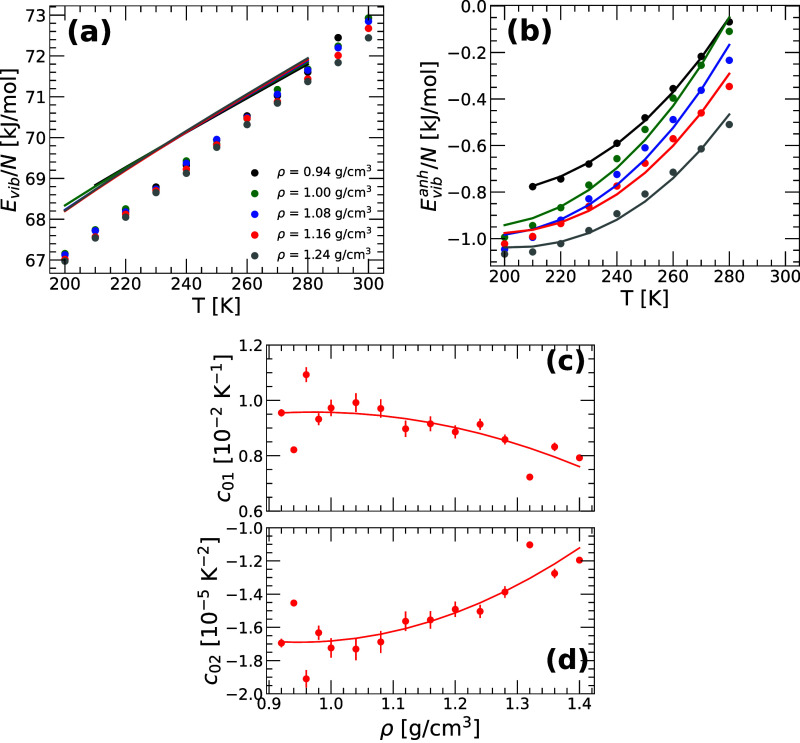
(a) Vibrational energy, *E*_vib_(*T*)= *E*(*T*) – *E*_IS_(*T*), as a function of temperature for q-TIP4P/F water at densities ρ = 0.94–1.24 g/cm^3^. Symbols are results from PI computer simulations; solid lines are the predictions from the PEL formalism using the harmonic approximation, eq 22. For all densities and temperatures, eq 22 overestimates *E*_vib_(*T*). (b) Anharmonic corrections to the vibrational energy, *E*_vib_^anh^(*T*), obtained from (a) (circles) along with the corresponding fit using eq 25 (lines). (c) and (d) PEL variables {*c*_0,1_(*V*), *c*_0,2_(*V*)} defined in eq 25 and obtained from the fits in (b). Lines are fits using a quadratic polynomial.

Figure 3b shows the anharmonic corrections, *E*_vib_^anh^(*T*) = *E*_vib_(*T*) – *E*_vib_^harm^(*T*), resulting from Figure 3a [circles] together with the corresponding fit using eq 25 [lines]. For all the densities studied, eq 25 reproduces the PIMD simulation data very well, showing that anharmonicities in the RP-PEL of q-TIP4P/F water can indeed be modeled using eqs 10 and 11, and *B̃*_1_ = 0. The PEL variables {*c*_0,1_(*V*), *c*_0,2_(*V*)} (*N* is constant) resulting from the fits in Figure 3b are shown in Figure 3c,d for all the volumes considered. While the values of *c*_0,1_(*V*) and *c*_0,2_(*V*) are somewhat scattered at low (ρ < 1.00 g/cm^3^) and high densities (ρ > 1.30 g/cm^3^), both PEL parameters vary slowly with *V*, implying that the anharmonicities of the PEL basins sampled by supercooled q-TIP4P/F water depend weakly on the density.

#### 4.2.2 Configurational Entropy–Finding *c*_0,0_(*V*)

To further validate the PEL formalism for q-TIP4P/F water, we show that its configurational entropy *S*_IS_(*T*), obtained numerically from PI computer simulations, obeys eq 5 for all the densities considered.

The lines in Figure 4a are the theoretical predictions for *S*_IS_(*T*) based on the PEL formalism, eq 5, with the PEL variables {α­(*V*), *E*_0_(*V*), σ^2^(*V*)} obtained in ref 25 using classical MD simulations (see, e.g., the blue symbols in Figure 3c,d). In eq 5, *E*_IS_(*T*) is substituted using eq 13 (in equilibrium, *e*_IS_ → *E*_IS_), with *B̃*_1_ = 0. The symbols in Figure 4a correspond to the *S*_IS_(*T*) obtained numerically from PI computer simulations and thermodynamic integration.^53,61^ The procedure to calculate *S*_IS_(*T*) numerically involves a few steps, and is discussed carefully in refs 53,61 . Briefly, within the PEL formalism,

$$S_{\mathrm{IS}}(N,V,T)=S(N,V,T)-S_{\mathrm{vib}}(N,V,T)$$ 26

where *S*(*T*) is the total entropy of the liquid and *S*_vib_(*N*, *V*, *T*) is the corresponding vibrational entropy. To calculate *S*_IS_(*T*), one obtains *S*(*T*) via thermodynamic integration, using PI computer simulations.^53^ The vibrational entropy is defined as *S*_vib_(*N*, *V*, *T*) = [*E*_vib_(*N*, *V*, *T*) – *F*_vib_(*N*, *V*, *T*)]/*T*; using the Gaussian approximation and modeling anharmonic contributions using eqs 10 and 11 (*B̃*_1_ ≈ 0), one finds that^53^

$$S_{\mathrm{vib}}(N,V,T)=9Nn_{b}k_{B}[1-\ln(\beta A_{0})]-k_{B}S+k_{B}\beta {\left(\frac{\partial S}{\partial \beta }\right)}_{N,V,E_{\mathrm{IS}}}+k_{B}\left[-{\tilde{B}}_{0}+\beta {\left(\frac{\partial {\tilde{B}}_{0}}{\partial \beta }\right)}_{N,V}\right]$$ 27

­(the vibrational and total entropy are shown in Figure S4 of the SM). It follows from eqs 26 and 27 that *S*_IS_(*T*) can be evaluated numerically up to the undefined variable *c*_0,0_(*N*, *V*) (see eq 11). The symbols in Figure 4a correspond to the resulting *S*_IS_(*T*) evaluated numerically for selected isochores. For each density, the value of *c*_0,0_(*V*) is chosen to maximize the overlap with the corresponding theoretical prediction (lines; eq 5). The remarkable agreement among the theoretical and numerical values of *S*_IS_(*T*), at all densities studied, further indicates that the PEL formalism provides a reliable thermodynamic description of (q-TIP4P/F) water. The resulting values of *c*_0,0_(*V*) are shown in Figure 4b.

**Figure 4:**
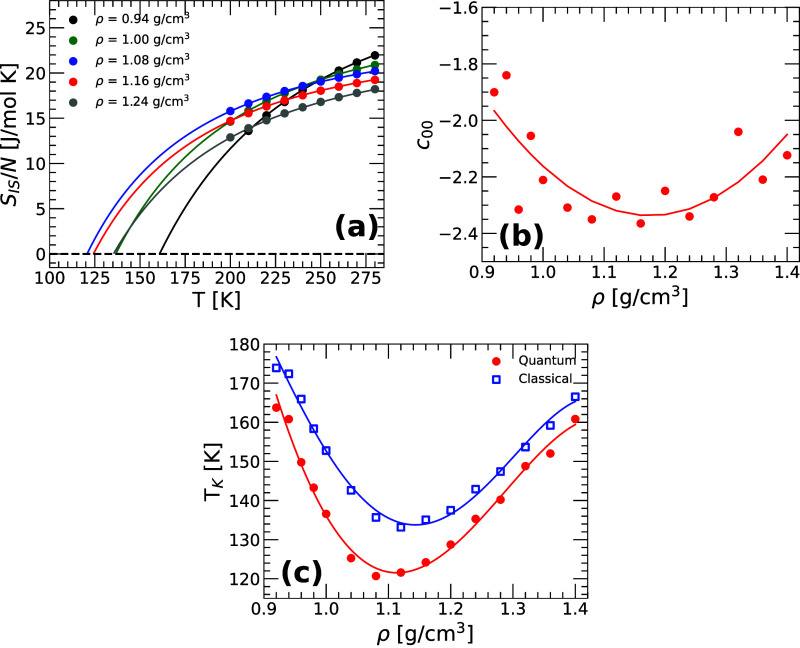
(a) Configurational entropy of q-TIP4P/F water *S*_IS_(*T*) as a function of temperature for selected densities in the range ρ = 0.94–1.24 g/cm^3^. The lines correspond to the theoretical prediction from the PEL formalism given in eq 5. The circles are the values of *S*_IS_(*T*) obtained numerically via thermodynamic integration and PI computer simulations. (b) The PEL variable *c*_0,0_(*V*) defined in eq 11 and obtained from (a); see text. The solid line is a fit using a quadratic polynomial. (c) Kauzmann temperature, *T*_*K*_(*V*), as a function of volume obtained by extrapolation of the lines in (a) to *S*_IS_(*T*_*K*_) ≡ 0 (red circles). For comparison, also included are the values of *T*_*K*_(*V*) obtained from the classical MD simulations of q-TIP4P/F water reported in ref^25^ (blue squares). For all the volumes studied, including NQE shifts *T*_*K*_(*V*) toward lower temperatures by 5–20 K, depending on the density considered. Lines are guides to the eye.

The temperature at which *S*_IS_(*N*, *V*, *T*) becomes zero is defined as the Kauzmann temperature, *T*_*K*_(*N*, *V*). It is a fundamental property in the study of liquids and glasses since it represent the temperature below which there is only one IS available to the system (liquid/glass).^46,69^ Figure 4c shows *T*_*K*_(*V*) (*N* is constant) for quantum (red circles; PI computer simulations) and classical q-TIP4P/F water (blue squares; MD simulations). The inclusion of NQE shifts *T*_*K*_(*V*) down by ≈5–20 K, with the largest changes occurring in the interval 0.98 ≤ ρ ≤ 1.16 g/cm^3^, encompassing the LLCP density (ρ_*c*_ ≈ 1.04 g/cm^3^; see Sec. [Sec sec4.3]).

### 4.3 Equation of State

To calculate the EOS of q-TIP4P/F water, we interpolate the *V*-dependent PEL variables in eqs 15–20, using polynomials. Specifically, {α­(*V*), *E*_0_(*V*), σ^2^(*V*)} are fitted to fourth-order polynomials; the corresponding interpolating lines are shown in Figures 2c,d^25^ and S1 in the SM. The variables {*c*_0,0_(*V*), *c*_0,1_(*V*), *c*_0,2_(*V*)} are fitted to a quadratic polynomial in *V*; see Figures 3c,d and 4b. The variables *a*(*T*, *V*) and *b*(*T*, *V*) depend on *T* and *V*; hence, they are expressed as sixth-order polynomials in *T*,

$$\begin{array}{ll} a(T,V)=\sum_{j=0}^{6}a_{j}(V)\;T^{j} & b(T,V)=\sum_{j=0}^{6}b_{j}(V)\;T^{j} \end{array}$$ 28

with *V*-dependent coefficients {*a*_*j*_(*V*)} and {*b*_*j*_(*V*)}. These coefficients are evaluated by first fitting *a*(*T*, *V*) and *b*(*T*, *V*) using eq 28 along each isochore. As shown in Figure S2 of the SM, these coefficients are smooth functions of *V*, and are interpolated using fourth-order polynomials in *V*.

Figure 5a compares the pressure *P*(*T*) obtained directly from the PI computer simulations along different isochores (circles) and the EOS predicted by the PEL formalism, eqs 15–20 (lines). The agreement between the PEL-EOS and PI computer simulations is very good over a wide range of densities, approximately 1.00 ≤ ρ ≤ 1.24 g/cm^3^. Importantly, this density range includes the LLCP density of q-TIP4P/F water (with electrostatic interactions treated using the reaction field technique), ρ_*c*_ ≈ 1.04 g/cm^3^ (see below). The deviations between the PI computers simulations and PEL-EOS at low (ρ < 1.00 g/cm^3^) and high densities (ρ > 1.24 g/cm^3^) may be explained, at least partially, by the noise in the *V*-dependence of some of the PEL variables involved; see, e.g. Figures 3c,d and 4b. Notably, even when the Gaussian approximation for the PEL of q-TIP4P/F water is valid up to *T* ≈ 280 K, the PEL-EOS holds above these temperatures.

**Figure 5:**
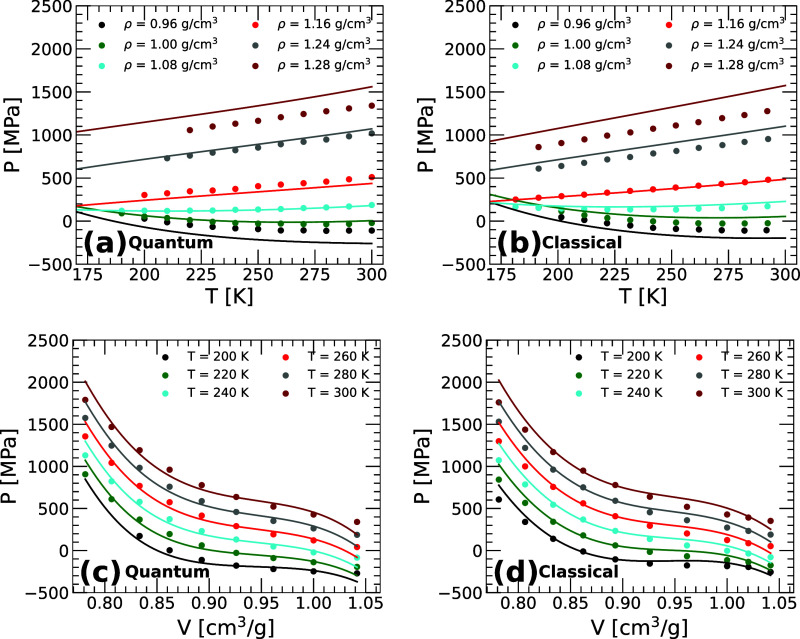
Isochores in the *P*–*T* plane for q-TIP4P/F water obtained from (a) PI computer simulations and (b) classical MD simulations (circles). Lines are the corresponding predictions based on the PEL-EOS (eqs 15–20 for quantum water; see ref 25 for classical water). The same set of isochores and temperature range is considered when calculating the PEL-EOS in (a) and (b). The PEL-EOS agrees very well with the pressures obtained directly from the MD/PI computer simulations at approximately 1.00 ≤ ρ ≤ 1.24 g/cm^3^, especially in the supercooled regime; deviations are observable at low and high densities. The quality of the PEL-EOS to reproduce the computer simulations results are comparable for quantum and classical (q-TIP4P/F) water. (c and d) Isotherms in the *P*–*V* plane for q-TIP4P/F water obtained from (c) PI computer simulations and (d) classical MD simulations, respectively (circles). Lines are the predictions from the corresponding PEL-EOS. For clarity, the isotherms *T* = 200, 220, 240, 260, 280, 300 K are shifted by Δ*P* = −300, −150, 0, 150, 300, 450 MPa.

The quantitative success of the PEL-EOS to reproduce the results from PIMD and MD simulations is comparable. To show this, included in Figure 5b is the PEL-EOS of classical q-TIP4P/F water. Importantly, for a fair comparison, the PEL-EOS in Figure 5a,b are both obtained by using the same isochores (0.96 ≤ ρ ≤ 1.24 g/cm^3^) and temperatures (200 ≤ *T* ≤ 280 K).^25^ The deviations between the computer simulation pressures (symbols) and the corresponding PEL-EOS (lines) are similar in Figure 5a,b. Similar conclusions follow from Figure 5c,d where the *P*(*V*)-isotherms of quantum and classical q-TIP4P/F water, from MD/PI computer simulations and the PEL-EOS, are compared to one another. It is expected that adding isochores and/or expanding the *T*-range in the determination of the PEL variables {*a*(*N*, *V*, *T*), *b*(*N*, *V*, *T*), *c*_0,0_(*N*, *V*), *c*_0,1_(*N*, *V*), *c*_0,2_(*N*, *V*)} will improve the agreement between the PEL-EOS and PI computer simulations (indeed, the PEL-EOS of *classical* q-TIP4P/F water improves, relative to the MD simulations, when additional densities are included; see ref 25).

To elucidate the NQE on the phase diagram of supercooled water, we compare in Figure 6a,b the *P*–*T* and ρ–*T* phase diagrams of quantum (solid lines) and classical (dashed-lines) q-TIP4P/F water. Included in these phase diagrams are the LLCP and associated binodal and spinodal lines, as well as the line of maximum density (ρ_max_) and Kauzmann temperature line (*T*_*K*_). Consistent with previous PI computer simulations, NQE shift the phase diagram of q-TIP4P/F water toward lower temperatures,^24,57,70,71^ and slightly lower pressures.^24,57^ This shift in *T* and *P* applies to the water isochores as well as the target properties reported in Figure 6a,b, i.e., the ρ_max_-line, *T*_*K*_-line, LLCP, binodal and spinodal lines. For a quantitative estimation of the observed NQE on the phase diagram of water, we focus on the location of the LLCP. The LLCP of q-TIP4P/F water obtained directly from the PI computer simulations is located at (ρ_*c*_ = 1.03 g/cm^3^, *T*_*c*_ = 180 K, *P*_*c*_ = 135 MPa) which is very close to the LLCP location predicted by the PEL-EOS, (ρ_*c*_ = 1.056 g/cm^3^, *T*_*c*_ = 187 K, *P*_*c*_ = 120 MPa). Similarly, the LLCP of classical q-TIP4P/F water is located at (ρ_*c*_ = 1.04 g/cm^3^, *T*_*c*_ = 190 K, *P*_*c*_ = 150 MPa), based on MD simulations, and (ρ_*c*_ = 1.058 g/cm^3^, *T*_*c*_ = 205 K, *P*_*c*_ = 169 MPa), based on the PEL-EOS of Figure 6b,d (as shown in ref 25, adding isochores to obtain the PEL-EOS of classical q-TIP4P/F water improves the agreement in the location of the LLCP between MD simulations and the PEL prediction). Based on the PEL-EOS of classical and quantum (q-TIP4P/F) water, it follows that NQE shift down the location of the LLCP of q-TIP4P/F water by approximately (Δρ_*c*_ = 0.002 g/cm^3^, Δ*T*_*c*_ = 18 K, Δ*P*_*c*_ = 49 MPa). The shift in the LLCP location is in semiquantitative agreement with previous estimations based on PIMD simulations and the two-state equation of state.^24,25^ Interestingly, the small differences in the values of (ρ_*c*_, *T*_*c*_, *P*_*c*_) for quantum and classical water obtained directly from PI/MD simulations and the corresponding PEL-EOS are comparable to one another.

**Figure 6:**
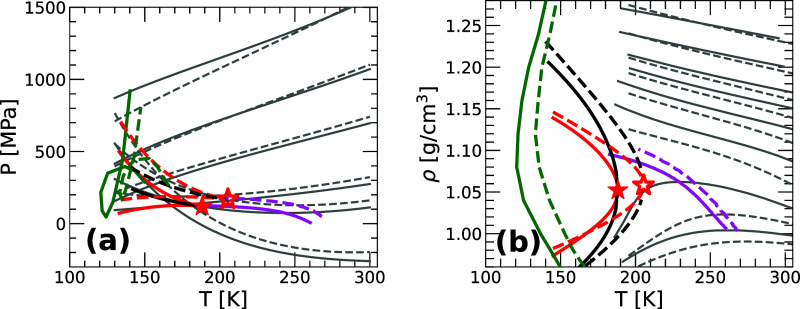
(a) *P*–*T* and (b) ρ–*T* phase diagrams of water predicted by the PEL-EOS for quantum (solid lines) and classical (dashed lines) q-TIP4P/F water. Included are the LLCP (red star), liquid–liquid binodal (black) and spinodal (red) lines. The magenta and green lines are the maximum density line and Kauzmann temperature, respectively. Gray lines in (a) are selected isochores (ρ = 0.96, 1.04, 1.12, 1.20, 1.24, 1.28 g/cm^3^, bottom to top), while in (b) gray lines correspond to selected isobars (*P* = 0.1, 100, 200, 300, 400, 500, 700, 1000 MPa, bottom to top). Including NQE shifts the LLCP location by (Δρ_*c*_ = 0.002 g/cm^3^, Δ*T*_*c*_ = 18 K, Δ*P*_*c*_ = 49 MPa).

We stress that the NQE on water are not limited to shifting its phase diagram in the *P*–*T* plane. As shown in Figure 6a, introducing NQE also alters the shape of the LDL-HDL coexistence region in the *P*–*T* plane, and affects the profiles of the ρ_max_-line and spinodal lines. Interestingly, the shape of the binodal line predicted by the PEL-EOS is barely altered by the inclusion of NQE. Similar nonuniform NQE were also observed in PI computer simulations of a water-like monatomic model liquid, where introducing quantum fluctuations not only shifted the phase diagram of the system, including the corresponding LLCP, but it also altered the slope and shape of the extrema lines in the *P*–*T* plane, including the ρ_max_-line.^70,71^

### 4.4 Adam–Gibbs Relation

One may expect that the dynamics of a liquid at low temperatures is influenced by the topography of the underlying PEL. Indeed, the Adam–Gibbs (AG) relation predicts that the diffusion coefficient *D* of a liquid and its *S*_IS_ are related as follows,

$$D=D_{0}\exp\left[-A/TS_{IS}\right]$$ 29

where *A* and *D*_0_ are *T*-independent quantities. The parameter *A* controls how fast *D*(*T*) decreases upon cooling, as *T* and *S*_IS_(*T*) decrease, and it has been related to the fragility of the liquid.^52^

Figure 7 shows a semilog plot of *D* versus 1/(*TS*_IS_) for q-TIP4P/F water. Results are based on RPMD simulations for selected isochores. The solid lines correspond to the fits of *D*(*T*) using eq 29. The agreement between the RPMD simulations and eq 29 is excellent for all the densities studied, indicating that despite the influence of NQE on the dynamics of water, the diffusivity remains governed by the topography of the quantum PEL. Interestingly, the parameter *A* increases with increasing density, suggesting that the fragility of water increases upon compression. Similar results were found in the case of a classical Lennard-Jones binary mixture.^52^

**Figure 7:**
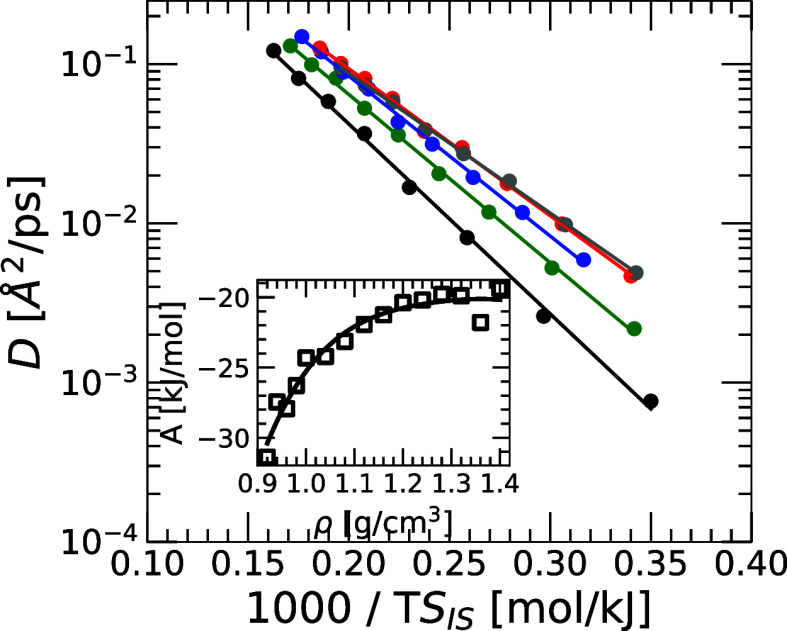
Diffusion coefficient of q-TIP4P/F water, *D*, as a function of 1000/*TS*_IS_ for selected densities ρ = 0.94, 1.00, 1.08, 1.16, 1.24 g/cm^3^ (bottom-to-top). The solid circles correspond to the values of *D* obtained from RPMD simulations; the lines correspond to the best fits using the Adam–Gibbs relationship (eq 29). For all the densities studied, the Adam–Gibbs relationship is in excellent agreement with the RPMD simulations, covering approximately 2 orders of magnitude in *D*. Inset: Parameter *A* defined in the Adam–Gibbs equation (eq 29) as a function of density.

## 5 Summary and Discussion

In this work, we combine the PEL formalism for quantum liquids^53,54,56,57,61^ and PI computer simulations to evaluate the equation of state of q-TIP4P/F water. Our results show that the PEL-EOS is in remarkable good agreement with the PI computer simulations over a wide range of temperatures and densities, approximately 185 < *T* < 300 K and 0.96 < ρ < 1.28 g/cm^3^, covering a pressure interval of ≈1000 MPa (Figure 5a,c). The quality of the PEL-EOS for quantum water (PI computer simulations) is comparable to the quality of the PEL-EOS obtained for classical water (MD simulations) when the *same* set of isochores and temperatures are considered (Figure 5). Adding isochores and/or expanding the temperature range may improve the overall performance of the PEL-EOS of quantum water.^25^

Our results based on the PEL-EOS for classical and quantum water show that including quantum fluctuations shifts the overall phase diagram toward lower temperatures and slightly lower pressures (Figure 6), consistent with previous PI computer simulations.^24,70–72^ In particular, the location of water’s LLCP shifts by Δ*T*_*c*_ ≈ 18 K, Δ*P*_*c*_ ≈ 49 MPa; the LLCP density remains rather unchanged, Δρ_*c*_ ≈ 0.002 g/cm^3^ (see also Table S3 of the SM). Interestingly, NQE also affect the shape of the liquid–liquid spinodal lines as well as the density-, and compressibility-maxima lines, consistent with PI computer simulations of water-like models.^70–72^ We note that the LLCP location estimated from the PEL-EOS, for both classical and quantum water, is consistent but slightly shifted down in *T* and up in *P* relative to the LLCP location estimated from experiments.^35,73^ For example, Mishima and Tatsuya used a large set of experimental volumes of water at different temperatures and pressures and provide an EOS for water.^74^ They find that the LLCP is located at about (*T*_*c*_ = 207 ± 5 K, *P*_*c*_ = 105 ± 9 MPa); we find (*T*_*c*_ = 185–188 K, *P*_*c*_ = 118–122 MPa) for q-TIP4P/F water based on PI simulations (NQE included) combined with the PEL formalism (see Table S3 of the SM). While the specific location of the LLCP varies slightly with the water model employed,^8^ we expect that the relative shift in the corresponding LLCP location upon the inclusion of NQE will be comparable to that found here for q-TIP4P/F water (Δ*T*_*c*_ ≈ 10–20 K, Δ*P*_*c*_ ≈ 15–50 MPa, Δρ_*c*_ ≈ 0.002–0.010 g/cm^3^).

In supercooled and liquid water, NQE are experimentally determined primarily through isotope substitution, H_2_O/D_2_O. Experiments suggest that including NQE, specifically, going from D_2_O to H_2_O, lowers the isothermal compressibility maximum temperature by 4 K at 1 atm.^14^ Similarly, the corresponding melting temperature and glass transition temperature of water decrease by 4 and 10 K, respectively, upon the D_2_O→H_2_O substitution.^37,41^ In addition, early experiments suggest that the LLCP temperature and pressure decrease upon isotope substitution, D_2_O→H_2_O.^36,73^ Our results are consistent with these findings. We find that including NQE, i.e., going from classical H_2_O (classical MD simulations) to quantum H_2_O (PI simulations) shifts the phase diagram of water to lower temperatures and pressures by approximately 18 K and 49 MPa. While we do not explicitly study D_2_O, the properties of water evolve monotonically along the sequence classical H_2_O→T_2_O→D_2_O→HDO→quantum H_2_O (see refs 38,53 ). Therefore, our PI simulations indicate that the phase diagram of water should shift to lower *T* and *P* upon isotope substitution D_2_O→H_2_O. Importantly, the shift in the LLCP temperature and pressure that we observe in q-TIP4P/F water (< 20 K and < 50 MPa for the classical H_2_O → quantum H_2_O substitution) are not inconsistent with the LLCP temperature/pressure shifts estimated from H_2_O/D_2_O experiments, 5–15 K and 30–70 MPa.^36,73^ In future work, it will be particularly interesting to apply the quantum PEL-EOS framework developed here to study the phase diagram of water isotopes in order to quantify how key thermodynamic properties, including the LLCP location, shift with the mass of the isotope.

Our PI simulations show that, as for the classical case, the PEL associated with q-TIP4P/F water is Gaussian (Figure 2a) with basins that are anharmonic (Figure 3). As shown in this work, at least for the q-TIP4P/F water model, anharmonic contributions can be modeled via eqs 10 and 11. The finding that the PEL variable *B̃*_1_ ≈ 0 indicates that the basins anharmonicities are rather independent of the corresponding IS energy, i.e., anharmonic contributions depend only on the temperature but not on the depth within the PEL at which the basin is located. The Gaussian character of the PEL implies that the configurational entropy *S*_IS_(*e*_IS_) of quantum water is given by a simple expression, eq 5. Moreover, the PEL parameters {α­(*V*), σ^2^(*V*), *E*_0_(*V*)} in eq 5 are identical to those of classical water. Therefore, the same expression for *S*_IS_(*e*_IS_) holds for classical and quantum water, and can be evaluated via classical MD simulations. This is because, as discussed in refs 53,54,56,57 , the ring-polymers associated with water O/H atoms are collapsed at the IS implying that the IS of classical and quantum water are isomorphic to one another, with identical IS energies.

An important property of supercooled liquids and glasses is the Kauzmann’s temperature, i.e., the temperature at which *S*_IS_(*T*) = 0. It follows that at *T* ≤ *T*_K_, there is only one (noncrystalline) IS in the PEL available to the liquid. Our PEL-based calculations indicate that, at all volumes considered, including NQE lowers the value of *T*_*K*_(*V*) of water and hence, shifts the corresponding *S*_IS_(*T*) toward lower temperatures (Figure 4a,c). The shift in *T*_K_(*V*) toward lower values (by ≈5–20 K, depending on the density) is an indication that NQE in water lowers the glass transition temperature. Indeed, as found in refs 24,38,64 , including NQE increases the diffusion coefficient of water, particularly at supercooled temperatures.

To explore the relationship between the topography of the PEL and the dynamics of quantum water, we also tested the Adam–Gibbs (AG) relation. Our PI simulations show that the AG relation holds for water in the presence of NQE, across all the isochores studied, covering approximately 2 orders of magnitude in *D*. As for classical water models, such as the SPC/E and TIP4*P*/2005 models,^75,76^ the diffusion coefficient of quantum q-TIP4P/F water is controlled by the number of accessible IS decreases via *S*_IS_ (and temperature) (Figure 7).

The PEL formalism is a general framework to study low-temperature liquids and glasses. As shown in this work, in the case of water, the PEL formalism for quantum liquids works remarkably well over a wide range of densities (0.96–1.28 g/cm^3^) and for approximately *T* < 280 K (although it predicts correctly many thermodynamics properties up to 300 K). From a more general perspective, the PEL formalism requires two *ansatzes*, for *S*_IS_ and *F*_vib_; with these two quantities, the Helmholtz free energy of the system *F*(*N*, *V*, *T*) is uniquely defined (eq 1). The most common assumptions in the PEL formalism for classical and quantum liquids that define *S*_IS_ and *F*_vib_ are the (i) Gaussian and (ii) harmonic approximations of the PEL (eqs 5 and 6). Unfortunately, it is not evident *a priori* whether the PEL of a given liquid is Gaussian and/or harmonic. This requires performing simple tests using computer simulations, as done here. Fortunately, condition (i) seems to be rather general and it holds for Lennard–Jones binary mixtures,^52,77^ atomistic models,^29,54,56^ and most realistic water models (including SPC/E,^42,76^ TIP4*P*/2005,^43^ q-TIP4P/F,^25^ and DP_MBpol^44^). Condition (ii) is not critical for the application of the PEL formalism since basins anharmonic corrections can always be included, as we showed in this work for the case of water.

In terms of computational time, RPMD simulations for a given system are approximately *n*_*b*_ times more expensive than the corresponding classical MD simulations. Hence, RPMD simulations become computationally more expensive, or may be inaccessible, at low temperatures (equilibration times increase upon cooling). In this regard, the PEL formalism is particularly useful since it provides an analytical expression for the Helmholtz free energy, including the PEL-EOS, that can be extrapolated to very low temperatures (*T* < 200 K), where RPMD simulations may not be equilibrated. As shown in this study, for the case of water, the PEL parameters needed for the Helmholtz free energy and PEL-EOS can be obtained from RPMD simulations at approximately *T* > 200 K; the PEL-EOS is reliable down to *T* < 185 K, below the LLCP temperature. We note that in the case of water, the PEL is Gaussian but anharmonic and hence, corrections to the Helmholtz free energy are necessary; such corrections must be evaluated from PI simulations. However, for quantum liquids characterized by a Gaussian and *harmonic* PEL, PI simulations are not really necessary. This is because, for a Gaussian and *harmonic* PEL, the Helmholtz free energy of the quantum liquid (eq 12) depends solely on parameters [α­(*V*), σ^2^(*V*), *E*_0_(*V*), {ω_*i*,0_^2^}_*i*=1,2,···,9*N*_] that are accessible from classical MD simulations. In such cases, the PEL formalism allows one to extract all of the thermodynamic properties of the quantum liquid of interest from classical MD simulations, avoiding the use of the more expensive RPMD simulations.

Overall, this work highlights the power of the PEL formalism to predict the thermodynamics and dynamics of low-temperature (classical and quantum) liquids, close to their glass transition temperature. The PEL formalism provides a relatively simple expression for the Helmholtz free energy *F*(*N*, *V*, *T*) that depends on a few PEL variables (8 variables, for the case of q-TIP4P/F water), all of them accessible from computer simulations, and many of them from classical MD simulations; the PEL-EOS follows from *F*(*N*, *V*, *T*) in a straightforward manner. It would be important in the future to test whether similar NQE apply to other liquids, including different water models. This is particularly important in the context of the LLCP location. Applying the PEL-based methodology introduced here to more accurate water models, such as MBpol, and machine-learned potentials based on quantum mechanical calculations, may provide a more reliable estimation of the role of NQE on the LLCP location.

## Supplementary Material



## Data Availability

The authors confirm that the data supporting the findings of this study are available within the article and its Supporting Information.

## Notes

Additional details for the calculation of the total, configurational, and vibrational entropy of q-TIP4P/F water based on thermodynamic integration and PI computer simulations; equilibration and production simulation times used at each state point; relevant parameters in the PEL-EOS; and an estimation of the uncertainties in the LLCP location of q-TIP4P/F water, demonstrating the robustness of the PEL-EOS relative to the (*T*, *V*)-states considered in the corresponding calculations (PDF)
