# Supplementary material for: Nuclear Quantum Effects on the Equation of State of Water: Insights from the Potential Energy Landscape Formalism
Source: J Chem Theory Comput. 2026 Mar 20;22(7):3208–21. doi: 10.1021/acs.jctc.5c02151 (PMC13085241; doi:10.1021/acs.jctc.5c02151)
Supplement: Supplementary file 1 [file ct5c02151_si_001.pdf]

Supplementary Material for 'Nuclear Quantum Effects on the Equation of State of Water: Insights from the Potential Energy Landscape Formalism'

Ali Eltareb[1,2,*], Gustavo E. Lopez[3,4,*], and Nicolas Giovambattista[1,2,4*]

[1] *Department of Physics, Brooklyn College of the City University of New York, Brooklyn, NY 11210, United States*

[2] *Ph.D. Program in Physics, The Graduate Center of the City University of New York, New York, NY 10016, United States*

[3] *Department of Chemistry, Lehman College of the City University of New York, Bronx, NY 10468, United States*

[4] *Ph.D. Program in Chemistry, The Graduate Center of the City University of New York, New York, NY 10016, United States*

---

[*] Electronic address: aeltareb@gradcenter.cuny.edu,gustavo.lopez1@lehman.cuny.edu,ngiovambattista@brooklyn.cuny.edu

I. COMPUTER SIMULATIONS

Table S1 shows the temperature and density of the state points studied using path-integral (PI) computer simulations of q-TIP4P/F water. The equilibration and production simulation times for each state point are also included.

**Densities 0.92–1.08 g/cm^3**				**Densities 1.12–1.40 g/cm^3**			
ρ	T [**K**]	t_{eq}	t_{pr}	ρ	T [**K**]	t_{eq}	t_{pr}
0.92	290–300	2.5	2.5	1.12	290–300	2.5	2.5
0.92	250–280	5	5	1.12	260–280	5	5
0.92	220–240	30	30	1.12	250	7.5	7.5
0.92	210	150	150	1.12	220–240	15	15
				1.12	210	20	20
				1.12	200	25	25
0.94	280–300	2.5	2.5	1.16	200	25	25
0.94	250–270	5	5	1.16	210	20	20
0.94	240	7.5	7.5	1.16	220–240	15	15
0.94	230	10	10	1.16	250	7.5	7.5
0.94	220	15	15	1.16	260–280	5	5
0.94	210	40	40	1.16	290–300	2.5	2.5
0.96	290–300	2.5	2.5	1.20	290–300	2.5	2.5
0.96	270–280	5	5	1.20	260–280	5	5
0.96	250–260	10	10	1.20	250	7.5	7.5
0.96	230–240	30	30	1.20	220–240	15	15
0.96	200–220	50	50	1.20	210	20	20
				1.20	200	25	25
0.98	280–300	2.5	2.5	1.24	290–300	2.5	2.5
0.98	250–270	5	5	1.24	260–280	5	5
0.98	240	7.5	7.5	1.24	250	7.5	7.5
0.98	230	10	10	1.24	220–240	15	15
0.98	220	15	15	1.24	210	20	20
0.98	210	20	20	1.24	200	30	30
0.98	200	40	40				
1.00	290–300	2.5	2.5	1.28	290–300	2.5	2.5
1.00	250–280	5	5	1.28	260–280	5	5
1.00	240	7.5	7.5	1.28	250	7.5	7.5
1.00	230	10	10	1.28	240	10	10
1.00	220	15	15	1.28	220–230	15	15
1.00	210	30	30	1.28	210	25	25
1.00	200	50	50				
1.00	190	50	50				
1.04	290–300	2.5	2.5	1.32	290–300	2.5	2.5
1.04	260–280	5	5	1.32	270–280	5	5
1.04	240–250	10	10	1.32	250–260	7.5	7.5
1.04	220–230	15	15	1.32	240	15	15
1.04	200–210	25	25	1.32	230	20	20
1.04	190	50	50				
1.08	290–300	2.5	2.5	1.36	290–300	2.5	2.5
1.08	260–280	5	5	1.36	270–280	5	5
1.08	250	7.5	7.5	1.36	250–260	7.5	7.5
1.08	220–240	15	15	1.36	240	15	15
1.08	200–210	25	25	1.36	230	20	20
1.08	190	50	50				
				1.40	290–300	2.5	2.5
				1.40	270–280	5	5
				1.40	250–260	7.5	7.5
				1.40	240	15	15
				1.40	230	20	20

TABLE S1: Equilibration (t_{eq}) and production (t_{pr}) times (ns) for all simulated densities and temperatures.

II. PARAMETERS IN THE PEL EQUATION-OF-STATE OF Q-TIP4P/F WATER

The PEL-EOS depends on the PEL variables $\{\alpha(V), E_0(V), \sigma^2(V), a(V,T), b(V,T), c_{0,0}(V), c_{0,1}(V), c_{0,2}(V)\}$ [Eqs. 15-20 of the main manuscript]. The parameters $\{E_0(V), \sigma^2(V), c_{0,0}(V), c_{0,1}(V), c_{0,2}(V)\}$ are given in Figs. 2(c)(d), 3(c)(d), and 4(b) of the main manuscript. As discussed in the main manuscript, the parameter $\alpha(V)$ is obtained from classical MD simulation and is shown in Fig. S1 (from Ref. [1]).

The coefficients $a(T,V)$, $b(T,V)$ (for a constant N) are given by Eq. 28 of the main manuscript and are defined by the T-independent coefficients $\{a_j(V)\}_{j=0,1,...6}$ and $\{b_j(V)\}_{j=0,1,...6}$. As shown in Fig. S2, these coefficients are smooth functions of V and can be fit very well with fourth-order polynomials.

It follows from Eqs. 15-20 and 28 of the main manuscript that the PEL-EOS for (q-TIP4P/F) water, $P(T,V)$, can be expressed analytically in terms of the following V-*dependent* PEL variables, $\{\alpha(V), E_0(V), \sigma^2(V), a_{j=0,1,..,6}(V), b_{j=0,1,..,6}(V), c_{0,0}(V), c_{0,1}(V), c_{0,2}(V)\}$ (N is constant). To evaluate the corresponding derivatives with respect to V in Eqs. 15-20, all these quantities are expressed as n-order polynomials in V with $n = 2$ or 4,

$$f(V) = \sum_{k=0}^{n} d_k V^k \qquad \text{(S1)}$$

The corresponding (constant) coefficients d_n are given in Table S2.

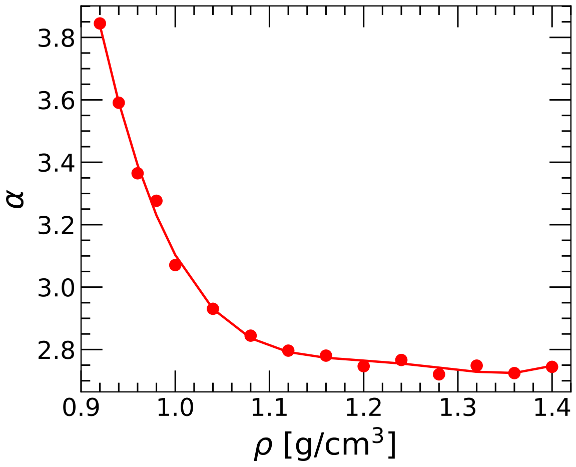

FIG. S1: PEL variable $\alpha(V)$ defined in Eq. 5 of the main manuscript as a function of volume; $\alpha(V)$ is obtained from classical MD simulations (from Ref. [1]). The line is a fit to the data using a fourth-order polynomial. The values of $\alpha(V)$ are the same for classical and quantum q-TIP4P/F water.

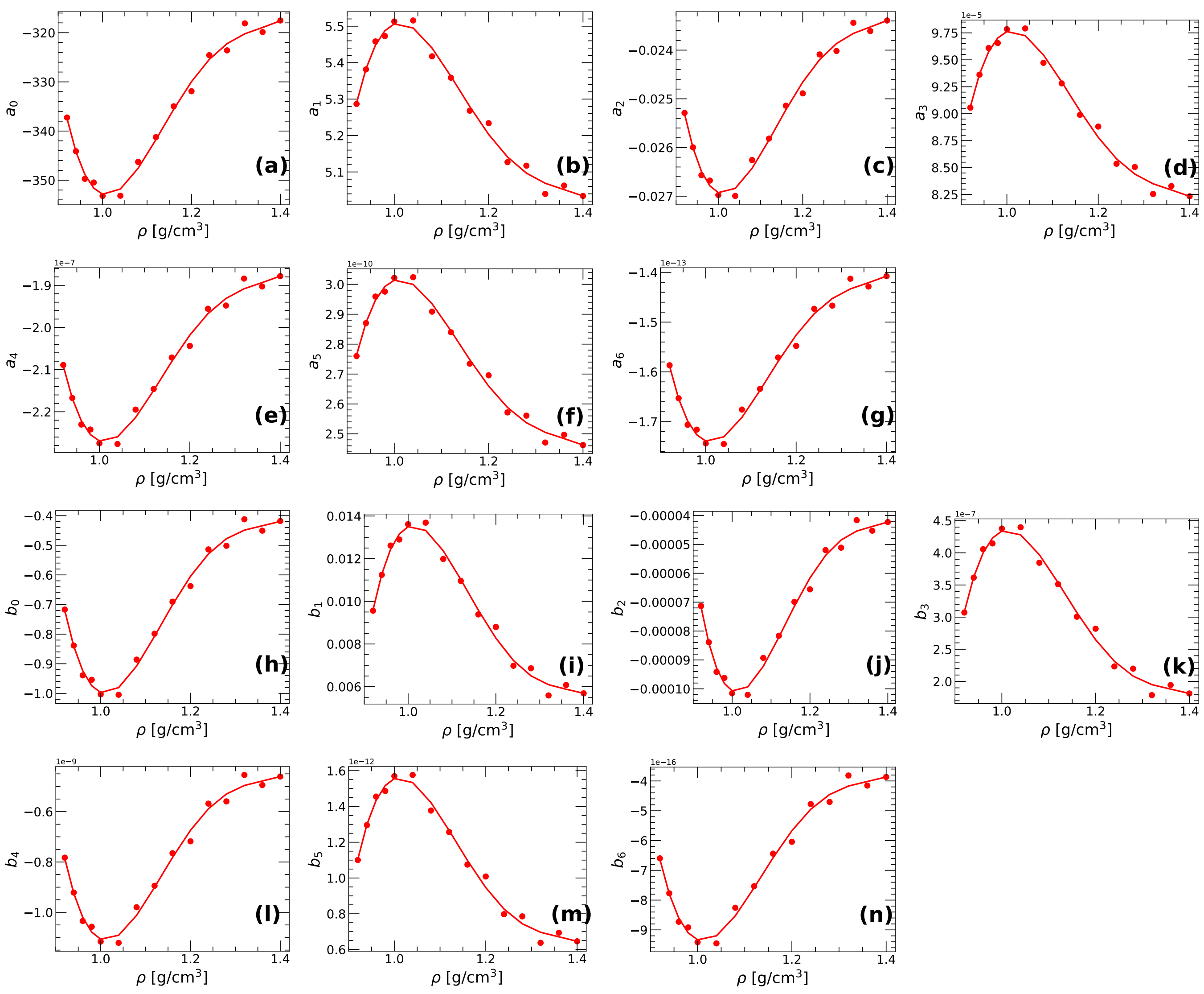

FIG. S2: Coefficients (a)-(g) $\{a_j(V)\}_{j=0,1,..,6}$ and (h)-(n) $\{b_j(V)\}_{j=0,1,..,6}$ defined in Eq. 28 of the main manuscript. These coefficients define the PEL variables $a(T,V)$ and $b(T,V)$ and hence, the shape function $\mathcal{S}$ of (q-TIP4P/F) water [Eq. 8 of the main manuscript]. All coefficients are smooth functions of V and can be interpolated very well using a fourth-order polynomials (lines; see Table S2).

	d_0	d_1	d_2	d_3	d_4
σ^2	-2.4030e+03	1.0758e+04	-1.7712e+04	1.2725e+04	-3.3527e+03
E_0	-5.7440e+01	4.8270e+01	-4.1884e+01	-7.0540e+01	6.9136e+01
α	-1.2749e+02	5.4661e+02	-8.4645e+02	5.7002e+02	-1.3959e+02
a_0	-5.3936e+03	2.0066e+04	-2.8439e+04	1.6821e+04	-3.4075e+03
a_1	5.6761e+01	-1.9576e+02	2.5819e+02	-1.3370e+02	2.0027e+01
a_2	-3.9756e-01	1.4069e+00	-1.8350e+00	9.2894e-01	-1.3022e-01
a_3	1.6735e-03	-5.9626e-03	7.7315e-03	-3.8666e-03	5.2188e-04
a_4	-4.2182e-06	1.5072e-05	-1.9477e-05	9.6706e-06	-1.2751e-06
a_5	5.8953e-09	-2.1100e-08	2.7210e-08	-1.3452e-08	1.7487e-09
a_6	-3.5195e-12	1.2608e-11	-1.6232e-11	7.9970e-12	-1.0275e-12
b_0	-9.8254e+01	3.8836e+02	-5.5494e+02	3.3336e+02	-6.9531e+01
b_1	1.0327e+00	-3.9318e+00	5.3007e+00	-2.8749e+00	4.8688e-01
b_2	-7.4927e-03	2.8365e-02	-3.7896e-02	2.0208e-02	-3.2852e-03
b_3	3.1891e-05	-1.2036e-04	1.6002e-04	-8.4540e-05	1.3426e-05
b_4	-8.0915e-08	3.0491e-07	-4.0435e-07	2.1260e-07	-3.3351e-08
b_5	1.1320e-10	-4.2593e-10	5.6352e-10	-2.9495e-10	4.5724e-11
b_6	-6.7890e-14	2.5536e-13	-3.3765e-13	1.7653e-13	-2.7290e-14
$c_{0,0}$	4.9032e-01	-6.5146e+00	3.7850e+00	–	–
$c_{0,1}$	1.3537e-02	-1.5015e-02	1.1420e-02	–	–
$c_{0,2}$	4.1003e-06	-2.9938e-05	8.3723e-06	–	–

TABLE S2: Coefficients $d_{k=0,1,...n}$ ($n = 2$ or 4) in Eq. S1 that define the PEL quantities $f(V) = \{\alpha(V), \sigma^2(V), E_0(V); a_{j=0,1,..,6}(V); b_{j=0,1,..,6}(V); c_{0,0}(V), c_{0,1}(V), c_{0,2}(V)\}$; see solid lines in Figs. 2(c)(d), 3(c)(d), 4(b) of the main manuscript and Fig. S1 and S2. Coefficients $\{d_k\}$ are given in units of the corresponding $f(V)$, over $(\mathrm{cm}^3/\mathrm{g})^k$.

	Quantum (PIMD)				Classical (MD)			
T **range [K]**	ρ_c [g/cm^3]	P_c [MPa]	T_c [K]	MSE [MPa^2]	ρ_c [g/cm^3]	P_c [MPa]	T_c [K]	MSE [MPa^2]
**200 - 280**	**1.056**	**120**	**187**	**5400**	**1.058**	**169**	**205**	**6697**
210 - 280	1.053	119	186	3001	-	-	-	-
220 - 280	1.048	118	185	1256	1.010	134	203	1970
230 - 280	1.060	122	188	7473	-	-	-	-
240 - 280	1.055	118	186	4967	-	-	-	-

TABLE S3: Location of the LLCP based on the PEL-EOS obtained using classical MD (from Ref. [1]) and PI computer simulations of q-TIP4P/F water performed at $0.96 \leq \rho \leq 1.28$ g/cm^3 and different temperature ranges. The mean-square error (MSE) is based on the differences between the pressure obtained from the PEL-EOS and the pressure calculated directly from the computer simulations. The location of the LLCP reported in the main manuscript are based on the temperature interval $T = 200 - 280$ K (first row). The location of the LLCP could not be determined from the classical MD simulations in Ref. [1] for the cases $T = 210 - 280$, $230 - 280$, and $240 - 280$ K.

III. UNCERTAINTIES IN THE LOCATION OF THE LLCP OF Q-TIP4P/F WATER

The PEL-EOS reported in the main manuscript for q-TIP4P/F water are based on PI computer simulations at densities $0.96 \leq \rho \leq 1.28$ g/cm^3 and $200 \leq T \leq 280$ K. The corresponding location of the LLCP predicted by the obtained PEL-EOS is $\rho_c = 1.056$ g/cm^3, $P_c = 120$ MPa, and $T_c = 187$ K. In order to test the robustness of the PEL-EOS to the range of temperature considered, we calculate the PEL-EOS using different temperature intervals. We focus on the location of the predicted LLCP. Table S3 includes the location of the LLCP based on the PEL-EOS and PI/MD simulations at $0.96 \leq \rho \leq 1.28$ g/cm^3 and different range of temperatures. In the quantum case, the LLCP location barely shifts with the T-interval considered; specifically, $\rho_c = 1.048 - 1.060$ g/cm^3, $P_c = 118 - 122$ MPa, and $T_c = 185 - 188$ K. In the classical case, our comparison is more limited since removing state points in the PEL analysis can make it difficult to locate the LLCP. In this case, we find $\rho_c = 1.010 - 1.058$ g/cm^3, $P_c = 134 - 169$ MPa, and $T_c = 203 - 205$ K. When the largest data sets form the PI/MD simulations are considered ($T = 200 - 280$ K), NQE shift the LLCP of q-TIP4P/F water down in density, temperature, and pressure by $\Delta\rho_c = 0.002$ g/cm^3, $\Delta P_c = 49$ MPa, and $\Delta T_c = 18$ K.

IV. ENTROPY OF Q-TIP4P/F WATER FROM THERMODYNAMIC INTEGRATION AND PI COMPUTER SIMULATIONS

In this section, we describe the procedure used to calculate the entropy of q-TIP4P/F water, $S(T,V)$ for a system composed of $N = 512$ water molecules. Briefly, $S(T,V)$ is obtained by thermodynamic integration using the expression $dS = \frac{1}{T}dE + \frac{P}{T}dV$, and the value for the entropy $S_0(T_0, V_0)$ at the reference state ($T_0 = 260$ K, $V_0 = 1.00$ cm^3/g).

(i) We first calculate the entropy of q-TIP4P/F water as a function of volume at the reference temperature T_0, $S(T = T_0, V)$. At $T = T_0$,

$$S(T_0, V) = S_0(T_0, V_0) + \frac{E(T_0, V) - E(T_0, V_0)}{T_0} + \frac{1}{T_0}\int_{V_0}^{V} P(T_0, V')dV' \tag{S2}$$

where $E(T,V)$ and $P(T,V)$ are the energy and pressure of q-TIP4P/F water at (T,V); both quantities can be obtained directly from the PI computer simulations [2]. To evaluate the integral in Eq. S2, the values of $P(T_0, V')$ are obtained from independent PI simulations performed at different volumes $V' = 0.72 - 1.09$ cm^3/g ($T = T_0$). As shown in Fig. S3(a), $P(T_0, V)$ is a smooth function of V and can be fitted very well with a fourth-order polynomial (line). This allows one to evaluate $S(T_0, V)$ analytically using Eq. S2. The energy and entropy of q-TIP4P/F water at the reference state point (T_0, V_0) are evaluated in our previous work [3] using PI simulations, $E_0(T_0, V_0) = 13.54$ kJ/mol and $S_0(T_0, V_0) = 55.40$ J/mol K.

(ii) Once the entropy at $T = T_0$ is evaluated for a given volume V using Eq. S2, we obtain the entropy at the target V as a function of temperature, $S(T,V)$ [in the rest of this section, we omit the V-dependence in all expressions]. At constant volume, $dE = TdS$ and hence,

$$S(T) = S(T_0) + \int_{T_0}^{T'} \frac{1}{T'}\left(\frac{\partial E}{\partial T}\right)_V dT' \tag{S3}$$

where $S(T_0)$ is the entropy given by Eq. S2 and $E(T)$ is the total energy of the system for the target isochore. To evaluate the integral in Eq. S3, we follow Ref. [3] and fit the values of the *total* energy $E(T)$ (obtained from independent PI simulations) using the following expression,

$$E(T) = a + bT^{3/5} \tag{S4}$$

where a and b are fitting parameters. As shown in Fig. S3(b), Eq. S4 holds remarkably well for the volumes considered.

Fig. S4(a) shows the values of $S(T)$ at selected volumes. As expected, $S(T)$ decreases monotonically upon cooling, and $S(T) > 0$ at all temperatures studied. We note that, as shown in Ref. [1], classical MD simulations of q-TIP4P/F water lead to unphysical results, with $S(T) < 0$ at low temperatures. This is a well-known problem inherent to classical statistical mechanics. In this regards, Fig. S4(a) highlights the importance of including NQE to restore thermodynamic consistency.

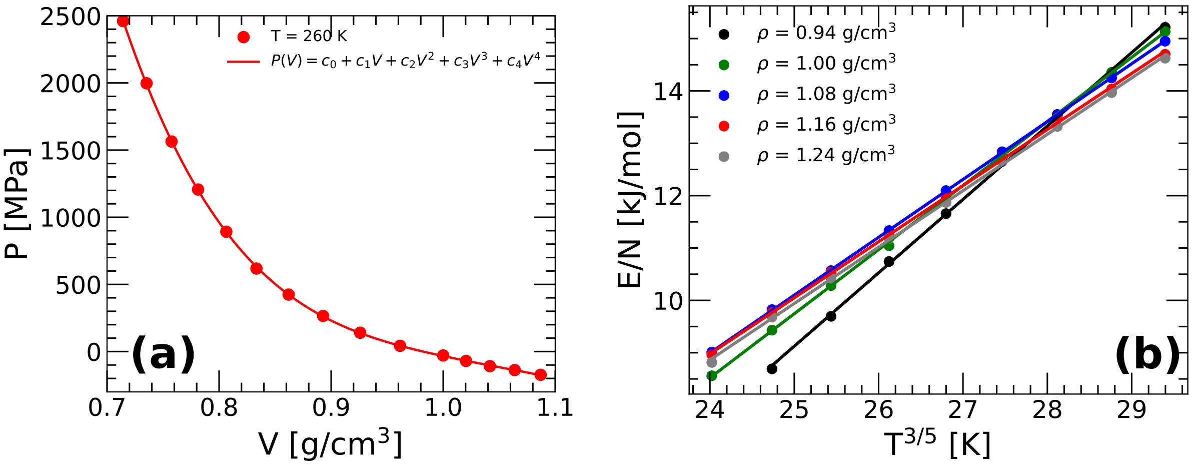

FIG. S3: (a) Pressure as a function of volume for q-TIP4P/F water obtained from PI computer simulations at the reference temperature $T_0 = 260$ K. The line is the corresponding fit to the data using a fourth-order polynomial. (b) *Total* energy $E(T)$ as a function of $T^{3/5}$ for q-TIP4P/F water along selected isochores. The circles are the values of $E(T)$ obtained from PI computer simulations; the lines are the corresponding fit using Eq. S4.

V. CONFIGURATIONAL AND VIBRATIONAL ENTROPY OF Q-TIP4P/F WATER

As we discussed in detail in Ref. [3], the total entropy of the system is given by

$$S(N,V,T) = S_{IS}(N,V,T) + S_{vib}(N,V,T) \tag{S5}$$

where the vibrational entropy is given by Eq. 27 of the main manuscript, and S_{IS} is the configurational entropy of the ring-polymer system associated to the quantum liquid studied (water). It can be shown that Eq. 27 can be rewritten as follows,

$$S_{vib}(N,V,T) = S_{vib}^{harm}(N,V,T) + S_{vib}^{anh}(N,V,T) \tag{S6}$$

where

$$S_{vib}^{harm}(N,V,T) = 9Nn_b k_B \left[1 - \ln(\beta A_0)\right] - k_B \mathcal{S} + k_B \beta \left(\frac{\partial \mathcal{S}}{\partial \beta}\right)_{N,V,E_{IS}} \tag{S7}$$

is the vibrational entropy in the harmonic approximation of the PEL, and

$$S_{vib}^{anh}(N,V,T) = k_B \left[-\tilde{B}_0 + \beta \left(\frac{\partial \tilde{B}_0}{\partial \beta}\right)_{N,V}\right] \tag{S8}$$

is the anharmonic contribution to the vibrational entropy. $S_{vib}^{harm}(N,V,T)$ and $S_{vib}^{anh}(N,V,T)$ must be calculated numerically using MD and PI simulations, respectively, in order to evaluate $S_{IS}(N,V,T)$ [3, 4].

Fig. S4(b) and S4(c) show the harmonic and anharmonic vibrational entropies, $S_{vib}^{harm}(T)$ and $S_{vib}^{anh}(T)$, at selected densities. We find that $S_{vib}^{harm}(T)$ is positive for all the temperatures and isochores studied, in contrast with the classical case where $S_{vib}^{harm}(T) < 0$ [1]. For comparison, included in Fig. S4(d) is the configurational entropy (Fig. S4(d) is a magnification of Fig. 4(a) of the main manuscript).

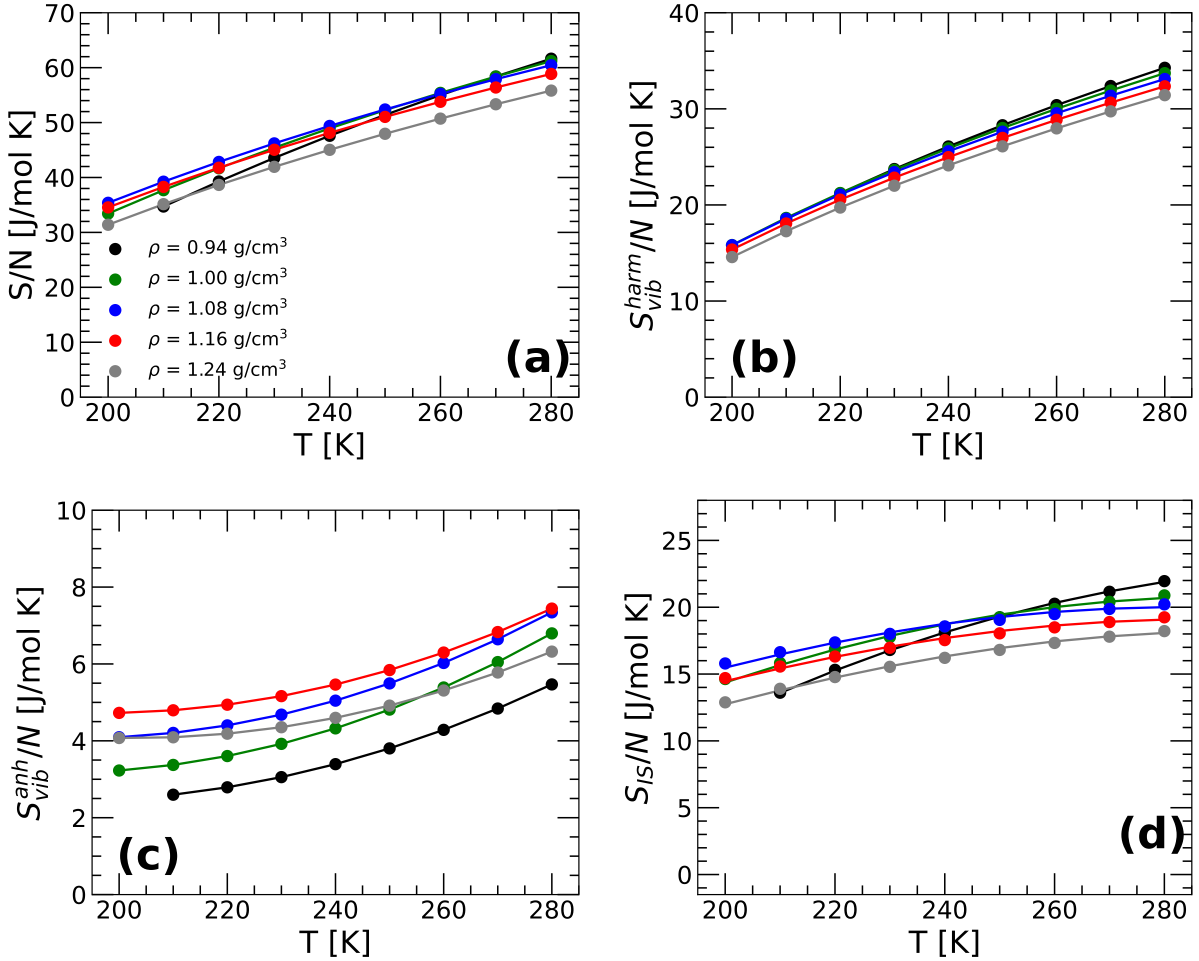

FIG. S4: (a) Entropy $S(T)$ of q-TIP4P/F water as a function of temperature obtained from thermodynamic integration and PI computer simulations [Sec. IV]. (b) Harmonic and (c) anharmonic contributions to the vibrational entropy, $S_{vib}^{harm}(T)$ and $S_{vib}^{anh}(T)$ [Eqs. S7 and S8]. (d) Configurational entropy $S_{IS}(T) = S(T) - S_{vib}^{harm}(T) - S_{vib}^{anh}(T)$ obtained numerically from (a)-(c) (symbols) and theoretically using Eq. 5 of the main manuscript (lines). Panel (d) is a magnification of Fig. 4(a) of the main manuscript.

[1] A. Eltareb, G. E. Lopez, and N. Giovambattista, J. Chem. Phys. **160**, 154510 (2024).
[2] M. Tuckerman, *Statistical mechanics: theory and molecular simulation* (Oxford University Press, 2010).
[3] A. Eltareb, Y. Zhou, G. E. Lopez, and N. Giovambattista, J. Chem. Theory Comput. **21**, 11931 (2025).
[4] Y. Zhou, A. Eltareb, G. E. Lopez, and N. Giovambattista, arXiv preprint arXiv:2507.21323 (2025).
